# Activation of estrogen-related receptor γ by calcium and cadmium

**DOI:** 10.3389/fendo.2024.1400022

**Published:** 2024-10-23

**Authors:** Qiaochu Wang, Nanxi Huang, John B. Psaltis, Reem M. Gahtani, Gai Yan, Dajun Lu, Shannon R. Cahalan, Xu Shi, Robert L. Copeland, Bassem R. Haddad, Mary Beth Martin

**Affiliations:** ^1^ Department of Biochemistry and Molecular and Cellular Biology, Georgetown University, Washington, DC, United States; ^2^ Oncology, Georgetown University, Washington DC, United States; ^3^ Department of Pharmacology, College of Medicine, Howard University, Washington, DC, United States

**Keywords:** estrogen-related receptor γ, gluconeogenesis, metals, calcium, cadmium

## Abstract

**Objective:**

Estrogen-related receptor γ (ERRγ) is a metabolic regulator with no identified physiological ligands. This study investigates whether calcium is an ERRγ ligand that mediates the effects of glucagon and whether cadmium, which mimics the effects of calcium, disrupts metabolism through ERRγ.

**Method:**

HepG2, MCF-7, and HEK293T transfected with ERRγ were treated with glucagon, calcium, cadmium, ERRγ agonist, or ERRγ inhibitor. Cells were then collected for *in vitro* assays including real-time qPCR, Western blot, ChIP, immunofluorescence, mutational analysis, or gene set enrichment analysis. Molecular dynamics simulations were performed to study mutation sites.

**Results:**

In HepG2 cells, treatment with glucagon, calcium, or cadmium re-localized ERRγ to the cell nucleus, recruited ERRγ to estrogen-related response elements, induced the expression of ERRγ-regulated genes, and increased extracellular glucose that was blocked by an ERRγ antagonist. In MCF-7 cells and HEK293T cells transfected with ERRγ, similar treatments induced the expression of metabolic genes. Mutational analysis identified S303, T429, and E452 in the ligand-binding domain as potential interaction sites. Molecular dynamics simulations showed that calcium induced changes in ERRγ similar to ERRγ agonist.

**Conclusion:**

The results suggest that calcium is a potential ligand of ERRγ that mediates the effects of glucagon and cadmium disrupts metabolism through ERRγ.

## Introduction

Disruption of metabolism is associated with many diseases, including obesity, type 2 diabetes, nonalcoholic fatty liver disease, and several cancers including breast, liver, and prostate cancers as well as many other medical conditions (1–4). The soaring rates of these associated diseases pose a big threat to society (5). For example, there are at least 400 million obese adults worldwide according to the World Health Organization, the number of which will only increase in the next decades (6). It is also predicted that worldwide, the number of patients with diabetes will rise from 171 million in 2011 to approximately 366 million in 2030 (7, 8). Genetics, lifestyle, and exposure to environmental toxins have been identified as risk factors for developing metabolic diseases; however, the mechanisms underlying the risk are not fully understood. There is increasing evidence linking exposure to environmental contaminants, such as bisphenol A (BPA), pesticides, and cadmium, to the disruption of metabolism in humans (3, 4). Several studies have linked cadmium with obesity and type 2 diabetes (9, 10). Cadmium is a heavy metal. Exposure to the metal occurs through industrial applications and agricultural products, such as food and tobacco, as a result of cadmium-contaminated soil and water. Our published results in rodents show that dietary exposure to environmentally relevant amounts of the metal cadmium or the metalloid arsenite increased body weight in a rodent model (11). In addition to cadmium, several studies demonstrate that the metal calcium is essential in metabolism, suggesting that alteration in calcium homeostasis or frequent exposure to some metals and metalloids disrupts cellular signaling and strongly influences metabolism (7, 9, 11–16).

The estrogen-related receptors (ERRs) are members of the superfamily of nuclear receptors that share similar structures and sequences to other nuclear receptors. There are three isoforms of ERRs: ERRα, ERRβ, and ERRγ. Although ERRs share high homology with estrogen receptor α (ERα), ERRs do not bind estrogens or participate in estrogen-regulated pathways (17). Instead, ERRα and ERRγ are involved in the regulation of metabolism and ERRβ is involved in stemness (18–20). Because of the important role of ERRγ in regulating metabolism, there has been an intensive research effort to identify natural ligands for ERRs. However, no natural ligands for the receptors have been identified so far but recent studies have identified a few synthetic ligands (19, 21). In glucose metabolism, ERRγ regulates the expression of the rate-limiting enzymes in the gluconeogenesis pathway including glucose-6-phosphatase (G6PC) and phosphoenolpyruvate carboxykinase (PEPCK) (22, 23). It also regulates pyruvate dehydrogenase kinase 4 (PDK4) that inhibits the pyruvate dehydrogenase complex (22, 23). One study suggests that ERRγ can be modified by glucagon through post-translational modification (24). However, the mechanism requires further investigation. In lipid metabolism, ERRγ regulates lipogenesis through sterol regulatory element-binding protein-1c in the liver (25). ERRγ also increases uncoupling protein 1 during brown adipogenesis and fatty acid oxidation in brown adipocytes (26). Moreover, ERRγ regulates the mitochondrial genetic network that coordinates the postnatal metabolic transition in the heart, which suggests that ERRγ plays a vital role in regulating the metabolic switch (27). Recent studies also reveal that ERRγ is involved in many metabolic diseases. For example, ERRγ was identified as a new obesity-susceptibility gene in an epigenomic study (28). The expression of ERRγ is elevated in patients with type 2 diabetes (29). Inhibition of ERRγ ameliorates diabetes and alcoholic fatty liver and rescues impaired insulin signaling, suggesting a role for ERRγ in metabolism under both physiological and pathological conditions (25, 30, 31).

Similar to other nuclear receptors, ERRγ is divided into several domains. ERRγ has an N-terminus transactivation function-1 (AF-1) domain that is involved in transcriptional activities, a DNA binding domain (DBD) that binds to estrogen-related response elements (ERRE), a hinge region that helps it fold into a three-dimensional structure, and a ligand-binding domain (LBD) that has the transactivation function-2 (AF-2) domain that interacts with coregulators (22). ERRγ has multiple splice variants, and these splice variants are tissue specific. For example, ERRγ splice variant 1 (ERRγ1) is regarded as the canonical isoform and accounts for 98.9% of ERRγ in placenta while ERRγ splice variant 2 (ERRγ2), which is missing the first 23 amino acids, is richer in tissue such as pancreas, prostate, and kidney (32). Although ERRγ regulates transcriptional activity as a monomer or homodimer, homodimerization enhances its transcriptional activity (33). Some studies suggest that ERRγ also forms a heterodimer with ERRα (33–35). Identifying natural and environmental ligands of ERRγ will help further our understanding of the role of ERRγ in metabolism and metabolic functions.

Our previous studies identified a new class of environmental estrogens, referred to as “metalloestrogens,” that activate ERα through a high-affinity interaction with its LBD (11, 36–40). Metalloestrogens include bivalent cations such as calcium, cadmium, and cobalt and anions such as arsenite, nitrite, and selenite (36). These studies show that calcium mediates the cross-talk between epidermal growth factor and ERα to induce ERα downstream regulated genes such as pS2 and progesterone receptor (37). The studies also show that an environmentally relevant dose of cadmium, which has similar properties to calcium, acts as an estrogen-like compound and activates ERα (11, 38, 39). The high degree of structural and amino acid homology between the LBDs of ERRs and ERα suggests that metals such as calcium and cadmium may function as ligands of ERRs and exposure to metals may influence metabolism.

This study asks whether calcium is a ligand of ERRγ that mediates the effect of glucagon on gluconeogenesis and whether environmental exposure to metals that mimic calcium, such as cadmium, disrupts glucose metabolism through ERRγ. The results show that calcium and cadmium increase the expression of genes involved in gluconeogenesis through an interaction with the LBD of ERRγ. In addition, potential interaction sites of calcium and cadmium with the LBD of ERRγ were identified, suggesting a potential role of calcium and cadmium as ligands of ERRγ.

## Materials and methods

### Reagents

Calcium chloride, cadmium chloride, and glucagon were purchased from Sigma-Aldrich. 4-Hydroxyn tamoxifen, ICI-182,780, 2-APB, DY131, and GSK4716 were purchased from Tocris.

### Cell culture

HEK293T cells (RRID: CVCL_0063) were a gift from Dr. Anna Riegel, Georgetown University. HepG2 cells (RRID: CVCL_0027) and MCF-7 cells (RRID: CVCL_0031) were acquired from Tissue Culture and Biobanking Shared Resource (Georgetown University). For cell passaging, HEK293T cells were maintained in improved minimal essential medium (IMEM; Corning) containing 10% fetal bovine serum (FBS; Sigma-Aldrich). HepG2 and MCF-7 cells were maintained in Dulbecco’s Modified Eagle’s Medium (DMEM; Corning) containing 10% FBS. For the assays, 5 × 10^5^) cells were plated in a six-well plate with 3 mL of lipoic acid-free IMEM (Crystalgen or VitaScientific) containing 5% charcoal-stripped calf serum (CCS; Sigma-Aldrich) for 48 h. The serum was diluted to 1% CCS. To increase the concentration of intracellular calcium, cells were treated with 1–10 mM calcium to activate calcium-sensing receptors. To obtain an intracellular concentration of cadmium similar to calcium, cells were treated with 5–10 µM cadmium. Cells were also treated with 100 nM glucagon, 1 µM DY131, or 1 µM GSK4716. All treatments were done in the absence or presence of 5 µM 4-hydroxy tamoxifen, 50 µM 2-APB, or 1 µM ICI-182,780 for 3 to 48 h.

### Real-time quantitative polymerase chain reaction

Following treatment, RNA was isolated using Trizol (Life Technologies). The reverse transcription reaction was performed using Maxima™ H Minus cDNA Synthesis Master Mix with dsDNase (ThermoFisher Scientific). qPCR assays were performed using Taqman probes (ThermoFisher Scientific), SsoAdvanced Universal Probes supermix (Bio-Rad), or PowerUp™ SYBR™ Green Master Mix (ThermoFisher Scientific). qPCR results were presented as fold change compared to control by using the 2^−ΔΔCt^ method and normalizing to the ribosomal protein P0 (RPLP0) mRNA (Taqman assays) or β-galactosidase mRNA (SyBr Green assay) (**Table 1**).

### Intracellular calcium measurement

HepG2 or HEK293T cells were plated in T75 flasks in IMEM with 5% CCS for 2 days and then cells were treated with 100 nM glucagon or 1–10 mM calcium in the absence or presence of 50 µM 2-APB for 3 to 24 h in IMEM with 1% CCS. After treatment, cells were trypsinized and washed with Hanks’ balanced salt solution (HBSS; Gibco) twice. Fluo-4 AM (15 µL; Invitrogen) was dissolved in 3 mL of HBSS and mixed with cells at 37°C for 30 min. Then, cells were washed with HBSS twice. Cells (5 × 10^5^) were used for fluorescence measurement by using the fluorimeter (Photon Technology International). The excitation wavelength was 494 nm and the emission wavelength was 516 nm. The first reading was labeled as F. Cells were then treated with 20 µL of Triton X-100 (Boston Bioscience) and the reading was labeled as *F*_max_. Cells were then treated with 40 µL of EGTA (Promega) and the reading was labeled as *F*_min_. The calcium concentration was calculated as follows: $Ca=K_{d}\;\times \;\left(F-F_{\text{min}}\right)÷\left(F_{\text{max}}-F\right)$ , where *K*_d_ is the affinity of the dye for calcium and its value is 335.

### Western blot

HepG2 cells were cultured in 100-mm dishes in IMEM with 5% CCS for 2 days and then treated with 100 nM glucagon, 10 mM calcium, or 5 µM cadmium for 3 h in IMEM with 1% CCS. Cells were then washed with phosphate-buffered saline (PBS) and lysed by radioimmunoprecipitation assay (RIPA) buffer. After removing cell debris by centrifuge, the protein concentration was determined by the Bio-Rad Protein Assay Dye Reagent (Bio-Rad Laboratories). Cell lysates were denatured by boiling and ran on 10% SDS-PAGE gel under reducing conditions, transferred to a 0.45-µm nitrocellulose membrane, blocked by 5% non-fat dry milk in PBS-Tween, and incubated with anti-PEPCK1 antibody (A2036, ABclonal, RRID: AB_2764060, 1:500), anti-G6PC antibody (ab93857, Abcam, RRID: AB_10903775, 1:200), anti-solute carrier family 2 member 2 (SLC2A2) antibody (ab192599, Abcam, 1:1,000), or anti-actin antibody (MAB1501, Millipore Sigma, RRID: AB_2223041, 1:2,000) at 4°C overnight and followed by anti-rabbit IgG (SA00001-2, Proteintech, RRID: AB_2722564, 1:5,000) or anti-mouse IgG (5220-0341, SeraCare, RRID: AB_2891080, 1:10,000) for 1 h at room temperature. The target protein signals were visualized using the Western Lightening^®^ Chemiluminescence Reagent Plus (Perkin-Elmer). The protein amount was measured by ImageJ and presented as fold change (± SEM) compared to control and normalized to the level of actin.

### Chromatin immunoprecipitation assay

HEK293T cells (3 × 10^6^) cells were plated in 150-mm culture dishes in IMEM with 5% CCS for 3 days. The HEK293T cells were then transfected with 25 µg of pSG5-HA-ERRγ or pCMX-hERRγ for 24 h. Cells were treated with 10 mM calcium or 10 µM cadmium in IMEM with 1% CCS for 1 h. HepG2 cells were grown in the same condition for 2 days, then treated with 100 nM glucagon, 10 mM calcium, or 10 µM cadmium in IMEM with 1% CCS for 1 h. Cells were fixed with 1% formaldehyde (ThermoFisher Scientific) for 5 min at room temperature, quenched by 125 mM glycine (Sigma-Aldrich) for 5 min, and washed with ice-cold PBS twice. Cells were collected by scraping and centrifugation, and then treated with nuclei isolation buffer (50 mM Tris-Cl, 60 mM KCl, 0.5% NP40, and 10 mM DTT) for 15 min on ice followed by centrifugation at 430 rcf for 10 min to pellet the nuclei. Isolated nuclei were then lysed with nuclei lysis buffer (EMD Millipore) on ice for 10 min, followed by flash-freezing in liquid nitrogen and thawing at room temperature to facilitate lysis. Chromatin was then sheared with the Bioruptor^®^ Plus sonicator (Diagenode) until the size was reduced to 300–500 bp. The soluble chromatin was then subjected to immunoprecipitation using anti-HA tag antibody (ab1424, Abcam, RRID: AB_301017) for PSG5-HA-ERRγ, and anti-ERRγ antibodies (pp-h6812, R&D, RRID: AB_604965) for pCMX-hERRγ or IgG (ab18413, Abcam, RRID: AB_2631983). Antibody/chromatin complexes were captured by Protein A/G magnetic beads (Thermofisher) on a magnetic rack, washed sequentially with low-salt buffer (EMD Millipore), high-salt buffer (EMD Millipore), LiCl buffer (EMD Millipore), and Tris-EDTA (Sigma-Aldrich), and eluted in chromatin immunoprecipitation assay (ChIP) elution buffer (1% SDS, 100 mM NaHCO_3_). Cross-link was reversed in 0.54 M NaCl overnight at 65°C. The samples were then treated with RNase (Fisher Scientific) and purified by the PCR purification kit (QIAGEN). DNA regions of interest were amplified by RT-PCR. To determine the potential ERRE sites of target genes, the UCSC Genome Browser was used. TNAAGGTCA as ERREs sequence template was used to find the potential ERRE of PEPCK1, G6PC, PDK4, and SLC2A2. The PDK4 ChIP primer sequence was previously published, and its ERRE location sequence was also verified in the UCSC Genome Browser (41).

### Immunofluorescence

HepG2 cells or MCF-7 cells were cultured on 18-mm circular cover slides in a 12-well plate in IMEM with 5% CCS for 2 days. For HepG2 cells, the serum was diluted to 1% on day 3 and cells were treated with 100 nM glucagon or 10 mM calcium for 3 h. For MCF-7, cells were grown in IMEM with 1% CCS for 24 h. Cells were then washed with PBS, fixed in 10% buffered formalin (Fisher Scientific) for 20 min and permeabilized using 0.5% Triton X-100 in PBS buffer for 5 min. Cells were then incubated in PBS containing 3% bovine serum albumin at room temperature for 40 min. Cells were treated with 1 µg/mL anti-ERRγ antibodies at 4°C overnight and then with anti-mouse polyclonal antibody (Alexa Fluor 594, Invitrogen, RRID: AB_2313921, 1:500) at room temperature for 60 min. F-actin was stained with Phalloidin-iFluor 488 Conjugate (AAT Bioquest, 1:500) for 60 min at room temperature. The nuclei were stained by 1 μg/mL 4,6-diamidino-2-phenylindole (DAPI, Boehringer Mannheim) for 5 min. The images were captured by Leica SP8.

### Glucose measurement

HepG2 cells were cultured in IMEM with 5% CCS for 2 days and then treated with 7.5 mM calcium, 10 mM calcium, 5 µM cadmium, or 10 µM cadmium in IMEM with 1% CCS for 24 h. Glucose concentration in the medium was measured by Glucose Assay Kit-WST (G264-05, dojindo).

### Chloramphenicol acetyltransferase reporter assay, mutagenesis assay, and transfection assay

PSG5-HA-ERRγ (ERRγ splice variant 1) was a gift from Dr. Rebecca Riggins, Georgetown University (42). pCMX-hERRγ (ERRγ splice variant 3) was a gift from Dr. Hongwu Chen, University of California, Davis (43). pCMV-GAL4 plasmid containing GAL4, pEQ176 plasmid containing β-galactosidase, and pG6 (5’Pro) plasmid containing chloramphenicol acetyltransferase (GAL-4-CAT) were purchased from Addgene. The amino acid sequences of the LBDs of ERRγ-1, -2, and -3 are identical. Because the sequences of the LBDs are the same, the LBD was subcloned from the ERRγ-1 construct. To create the GAL-4-ERRγ-LBD construct, the hinge region and LBD (L187-V458) of ERRγ from pSG5-HA-ERRγ was fused with the GAL-4 DNA binding domain from pCMV-GAL4 to form GAL-4-ERRγ-LBD. Restriction enzyme cut sites SacI were created in the 5′ terminus and XbaI was created in the 3′ terminus. Then, corresponding enzymes were used to fuse GAL-4-ERRγ-LBD with the empty pCMV vector. To create GAL-4-ERRγ-LBD mutants, mutation primers were designed using the QuikChange Primer Design of Agilent and synthesized by Integrated DNA Technologies. The GAL-4-ERRγ-LBD plasmid was mutated using the QuikChange Lightning Site-Directed Mutagenesis Kit (Agilent), and results were verified by sequencing. For the transfection assay, cells were transfected with 2.5 μg of pSG5-HA-ERRγ or pCMX-hERRγ using Trans-IT LT-1 (MirusBio) or SuperFect (QIAGEN) for 24 h. For the CAT reporter assay, cells were transfected with 2 μg of wild-type GAL-4-ERRγ-LBD or GAL-4-ERRγ-LBD mutant, 2 μg of pG6 (5’Pro), and 1 μg of pEQ176 using Trans-IT LT-1 for 24 h.

### Gene set enrichment analysis

Raw microarray data (accession no. GSE136595) were downloaded from Gene Expression Omnibus. Analysis of microarray data was performed by gene set enrichment analysis (GSEA) v4.1.0 for Windows. The hallmark gene sets and ontology gene sets from the Molecular Signatures Database were used in our analysis.

### Molecular dynamics simulations

The structure of ERRγ was obtained from Protein Data Bank (ID: 2GP7). The A chain of 2GP7 was then submitted to the Metal Ion-Binding Site Prediction and Docking Server (MIB; http://bioinfo.cmu.edu.tw/MIB/) or Autodock Vina to predict the corresponding calcium docking site (44, 45). All molecular dynamics simulations were performed by using QwikMD in VMD and the CHARMM36 force field. During the molecular dynamics simulations, the temperature of 37°C was set over a period of 1 ns. Resolution was set as Max, then solvent was set as Explicit, and other parameters were set based on the instruction of QwikMD. All analyses were performed by using the VMD plug-in (46). Graphs were created by PyMOL.

### Statistical analysis

All statistical analyses were performed in Prism. Data are presented as the mean ± standard error of the mean (SEM). Statistical differences were evaluated by one-way analysis of variance (ANOVA) followed by Fisher’s LSD test or *t*-test. Statistical significance is defined as a *p*-value of ≤0.05. *p ≤ 0.05; **p ≤ 0.01; ***p ≤ 0.001; ****p ≤ 0.0001.

## Results

### Effects of glucagon, calcium, and cadmium on the expression of ERRγ-regulated genes in HepG2 cells

**Figure 1:**
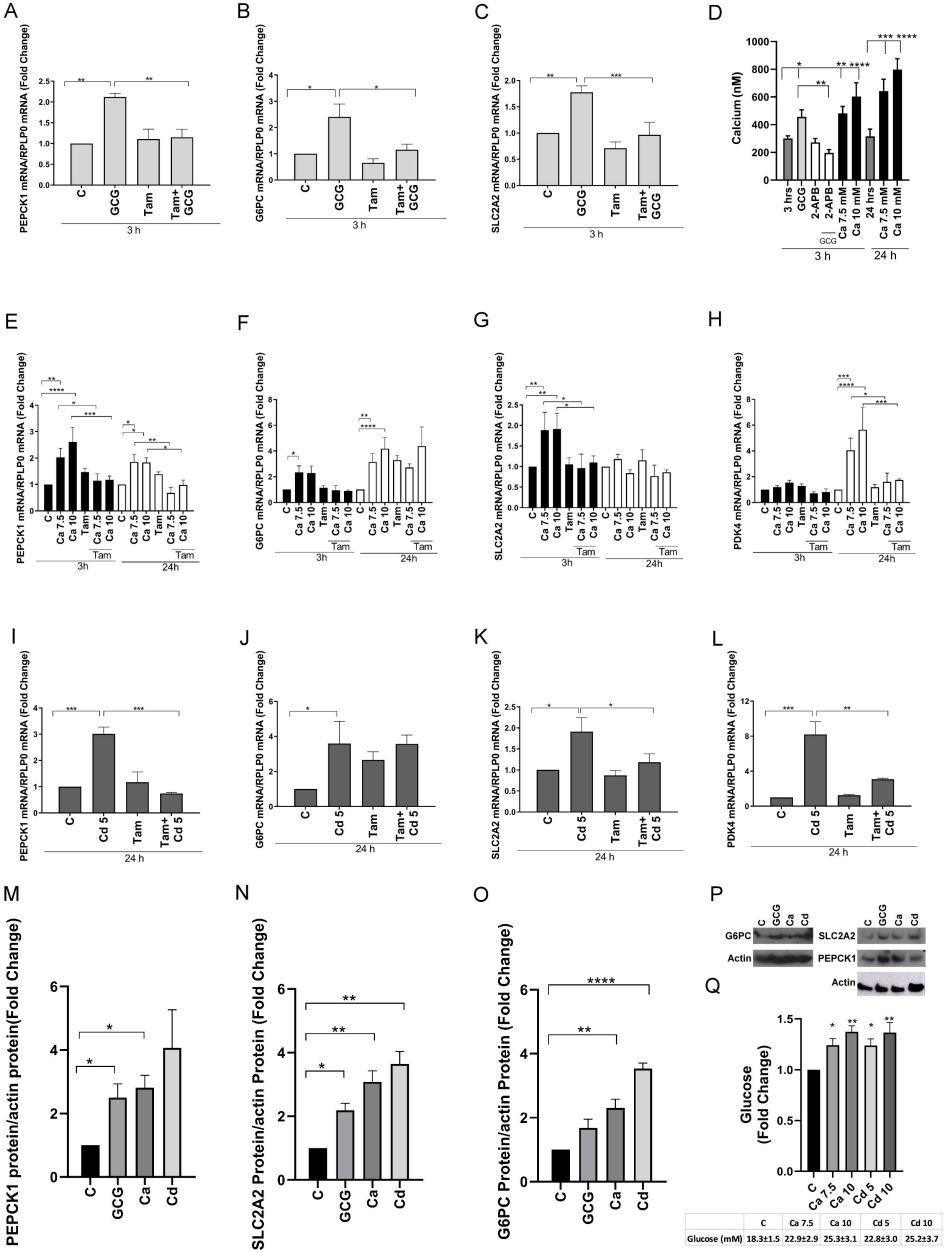
Effects of glucagon, calcium, and cadmium on ERRγ-regulated genes in HepG2 cells. HepG2 cells were treated with glucagon (GCG; 100 nM) for 3 h, calcium (Ca; 7.5 or 10 mM), or cadmium (Cd; 5 μM) for 3 and 24 h in the absence or presence of 4-hydroxy tamoxifen (Tam; 5 μM) or 2-APB (50 μM). The expression of PEPCK1, G6PC, SLC2A2, and PDK4 and the concentration of intracellular calcium were measured. For mRNA measurement, RNA was isolated and the amount of PEPCK1, G6PC, SLC2A2, and PDK4 mRNA was measured using a quantitative real-time-qPCR assay, normalized to the expression of ribosomal large protein P0 (RPLP0) mRNA, and presented as fold change. To quantify the concentration of intracellular calcium, the cells were trypsinized and incubated for 30 min with 5 μM Fluo-4 AM. The emission at 516 nm was determined using an excitation wavelength of 494 nm, and the concentration of intracellular calcium was calculated using the equation: [Ca_in_^2+^] = *K*_d_ × (*F* – *F*_min_)/(*F*_max_ – *F*), where *K*_d_ is the affinity of the dye for calcium. For protein measurement, PEPCK1, G6PC, and SLC2A2 were measured using Western blot, normalized to actin, and presented as fold change. Glucose in the medium was measured using a glucose assay kit. **(A–C)** Effects of glucagon on the induction of PEPCK1, G6PC, and SLC2A2 mRNA. **(D)** Effects of glucagon and extracellular calcium on the concentration of intracellular calcium. **(E–H)** Effects of calcium on the induction of PEPCK1, G6PC, SLC2A2, and PDK4 mRNA. **(I–L)** Effects of cadmium on the induction of PEPCK1, G6PC, SLC2A2, and PDK4 mRNA. **(M–P)** Effects of glucagon, calcium, and cadmium on the expression of PEPCK1, G6PC, and SLC2A2; representative images and quantitative analysis of Western blots. **(Q)** Effects of calcium and cadmium on glucose. Data are expressed as fold change (mean ± SEM); *n* ≥ 3. Statistical significance is defined as a *p*-value of ≤0.05. *p ≤ 0.05; **p ≤ 0.01; ***p ≤ 0.001; ****p ≤ 0.0001.

To determine whether calcium can mediate the cross-talk between glucagon and ERRγ, HepG2 cells were treated with glucagon (100 nM) or calcium (7.5 or 10 mM) in the absence or presence of the ERRγ inhibitor 4-hydroxy tamoxifen (5 μM). Treatment with glucagon for 3 h resulted in an approximately 2.1-, 2.4-, and 1.7-fold increase in the expression of PEPCK1, G6PC, and SLC2A2, respectively, which was significant and blocked by the ERRγ antagonist 4-hydroxy tamoxifen (**Figures 1A–C**). There was no significant effect on the expression of PDK4, which is not part of the gluconeogenesis pathway (data not shown). Similar to treatment with glucagon, treatment with calcium for 3 h significantly increased expression of PEPCK1, G6PC, and SLC2A2 by 2.6-, 2.3-, and 1.9-fold, respectively, which was blocked by 4-hydroxy tamoxifen. Treatment with calcium for 24 h resulted in a significant increase in the expression of PEPCK1, G6PC, and PDK4 by approximately 1.8-, 4.1-, and 5.6-fold, respectively (**Figures 1E–H**). 4-Hydroxy tamoxifen blocked the effects of calcium on PEPCK1 and PDK4 but failed to block the effects of calcium on G6PC and increased its expression. There is some evidence suggesting that 4-hydroxy tamoxifen can influence metabolism, which may be due to its effect on the expression of G6PC (47, 48).

Glucagon increases intracellular calcium through the inositol 1,4,5 trisphosphate receptor (IP3R)-mediated release of calcium from the endoplasmic reticulum, whereas high levels of extracellular calcium can increase intracellular calcium through the activation of calcium receptors and channels (49, 50). To determine whether intracellular calcium mediates the effects of glucagon and extracellular calcium on the activity of ERRγ, the concentration of intracellular calcium was measured following treatment with glucagon (100 nM) in the absence or presence of the IP3R inhibitor 2-APB (50 μM). The concentration of intracellular calcium was also measured following treatment with extracellular calcium (7.5 or 10 mM) (**Figure 1D**). As expected, treatment of HepG2 cells with glucagon for 3 h increased the concentration of intracellular calcium from approximately 300 (± 18) nM to 456 (± 51) nM that was blocked by 2-APB (196 ± 24 nM). Treatment with increasing concentrations of calcium for 3 h also increased the concentration of intracellular calcium to approximately 481 (± 50) to 602 (± 99) nM and treatment with increasing concentrations of calcium for 24 h further increased intracellular calcium concentration from 315 (± 53) nM to approximately 642 (± 85) to 797 (± 78) nM.

To determine whether cadmium mimics the effects of calcium on the activation of ERRγ and disrupts the glucagon signaling pathway, HepG2 cells were treated with cadmium (5 μM) for 24 h. Treatment with cadmium resulted in a significant increase of approximately 3.0-, 3.5-, 1.9-, and 8.1-fold in the expression of PEPCK1, G6PC, SLC2A2, and PDK4, respectively (**Figures 1I–L**). Similar to glucagon and calcium, 4-hydroxy tamoxifen blocked the effects of cadmium on PEPCK1, SLC2A2, and PDK4 but not the effect on G6PC.

To determine whether treatment with glucagon, calcium, or cadmium increased protein expression, the amount of PEPCK1, G6PC, and SLC2A2 was measured by Western blot analysis. Similar to the increase in mRNA, treatment with glucagon resulted in a significant 2.5- and 2.1-fold increase in PEPCK1 and SLC2A2, respectively. Treatment with calcium resulted in a 2.8-, 2.3-, and 3.0-fold increase in PEPCK1, G6PC, and SLC2A2, respectively, and treatment with cadmium resulted in a 3.5- and 3.6-fold increase in G6PC and SLC2A2, respectively, which were significant (**Figures 1M–P**).

To determine whether treatment with calcium and cadmium increased the concentration of glucose, the amount of glucose in the medium was measured. Treatment of HepG2 with calcium or cadmium for 24 h also significantly increased the concentration of glucose in the medium from 18 (± 1) mM to approximately 22 (± 2) to 25 (± 3) mM (**Figure 1Q**). Taken together, the results suggest that glucagon increases the expression of genes involved in gluconeogenesis through an increase in intracellular calcium and the subsequent activation of ERRγ and metals that mimic calcium increase the activity of ERRγ.

### Effects of glucagon, calcium, and cadmium on the recruitment of ERRγ to the enhancers of ERRγ target genes

**Figure 2:**
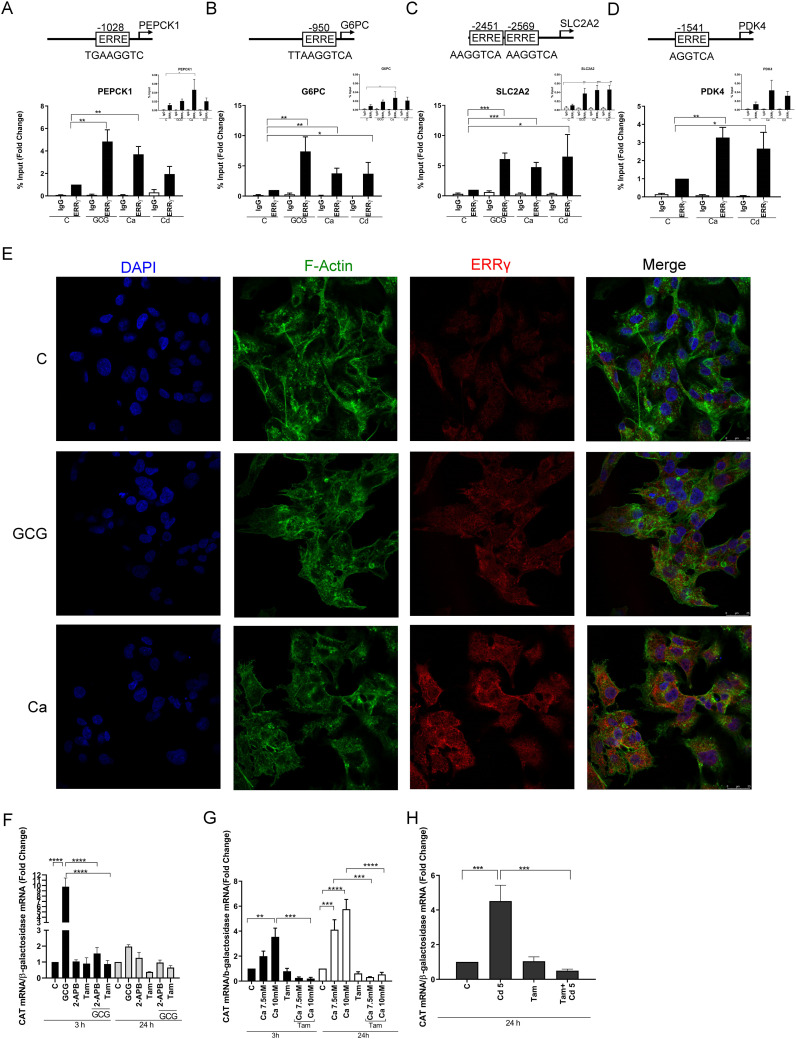
Effects of glucagon, calcium, and cadmium on the recruitment of ERRγ to the enhancers of ERRγ-regulated genes in HepG2 cells. HepG2 cells were treated with glucagon (GCG; 100 nM), calcium (Ca; 7.5 or 10 mM), or cadmium (Cd; 5 or 10 μM) for 1–24 h in the absence or presence of 4-hydroxy tamoxifen (Tam; 5 μM) or 2-APB (50 μM). To quantify the occupancy of ERRγ on the ERREs of target genes, HepG2 cells were treated for 1 h. The recruitment of ERRγ to the enhancers of target genes was measured using a quantitative ChIP-qPCR assay, and presented both as fold change over control of the % input values (main graphs) and as % input (insert graphs). To visualize the location of ERRγ, HepG2 cells were treated for 3 h and ERRγ localization was captured by immunofluorescence. To study the interaction between glucagon, calcium, or cadmium and ERRγ, HepG2 cells were transfected with the chimeric receptor GAL-4-ERRγ-LBD and a CAT reporter construct, treated for 3 to 24 h, and RNA was isolated. The amount of CAT mRNA was measured using a quantitative real-time qPCR assay, normalized to the expression of β-galactosidase mRNA, and presented as fold change. **(A–D)** Effects of glucagon, calcium, and cadmium on recruitment of ERRγ to the enhancer of PEPCK1, G6PC, SLC2A2, and PDK4. Figures illustrate the location of ERRE in enhancer regions. **(E)** Effects of glucagon and calcium on intracellular ERRγ localization. **(F–H)** Effects of glucagon, calcium, and cadmium induction of GAL4-coupled ERRγ LBD on expression of CAT reporter. Data are expressed as fold change (mean ± SEM); *n* ≥ 3. Statistical significance is defined as a *p*-value of ≤0.05. *p ≤ 0.05; **p ≤ 0.01; ***p ≤ 0.001; ****p ≤ 0.0001.

To further show that the change in gene expression is due to the activation of ERRγ by glucagon (100 nM), calcium (7.5 or 10 mM), and cadmium (5 or 10 μM), the recruitment of ERRγ to the enhancer regions of PEPCK1, G6PC, SLC2A2, and PDK4 was measured by a ChIP-qPCR assay. ERRγ is recruited to the ERR response element (TNAAGGTCA) in the enhancer regions of target genes and regulates gene expression. Through database searches, potential ERREs were identified in the enhancer region of the target genes (**Figures 2A–D**). Treatment with glucagon resulted in a significant enrichment of approximately 4.8-, 7.3-, and 6.0-fold of ERRγ on the enhancer regions of PEPCK1, G6PC, and SLC2A2, respectively. Treatment with calcium resulted in an approximately 3.7-, 3.7-, 4.7-, and 3.2-fold enrichment to the enhancer regions of PEPCK1, G6PC, SLC2A2, and PDK4, respectively, and treatment with cadmium resulted in an approximately 3.7-, 6.5-, and 2.6-fold enrichment to the enhancer regions of G6PC, SLC2A2, and PDK4, respectively, which were significant.

To determine the effects on the localization of ERRγ, HepG2 cells were treated with glucagon or calcium and the effects on ERRγ were measured by immunofluorescence. In control cells, ERRγ was localized to the periphery of the cell nucleus. Following treatment with glucagon or calcium, ERRγ re-localized in the nucleus (**Figure 2E**). Taken together, the results suggest that glucagon, calcium, and cadmium activate and recruit ERRγ to the ERRE in the enhancer regions of genes involved in gluconeogenesis.

### Identify the calcium and cadmium interaction domain on ERRγ

Mutational analysis and molecular modeling show that calcium interacts with the LBD of ERα to activate the receptor (37). The high homology between the LBDs of ERα and ERRγ suggests that the LBD of ERRγ contains potential calcium and cadmium interaction sites. ERRγ-1 is the canonical isoform of the receptor and shares the same LBD with other ERRγ splice variants. The LBDs of the splice variants ERRγ-1, -2, and -3 are identical. Calmodulin binds to ERRγ through two binding motifs (51). One calmodulin binding motif is located at the boundary between the N-terminus and DNA binding domain, and the other binding motif is located at the boundary of the hinge region and the N-terminus of the LBD. Both motifs are needed for calmodulin binding to ERRγ. To address the mechanism by which calcium and cadmium activate ERRγ, HepG2 cells were transfected with a chimeric receptor that contains the DNA binding domain of GAL4 and the LBD of ERRγ (GAL-4-ERRγ-LBD) and a CAT reporter construct. Following transfection, the cells were treated with glucagon (100 nM), calcium (7.5 or 10 mM), or cadmium (5 μM) in the absence or presence of the IP3 receptor inhibitor 2-APB (50 μM) or 4-hydroxy tamoxifen (5 μM) (**Figures 2F–H**). Treatment with glucagon for 3 h resulted in a significant increase of approximately 9.7-fold in the expression of CAT that was blocked by 2-APB and 4-hydroxy tamoxifen. Treatment with calcium for 3 and 24 h resulted in a significant increase of approximately 1.9- to 5.7-fold in the expression of CAT that was also blocked by 4-hydroxy tamoxifen. Similar to calcium, treatment with cadmium for 24 h resulted in a significant 4.5-fold increase of CAT that was blocked by 4-hydroxy tamoxifen. Taken together, the results suggest that calcium mediates the effects of glucagon through the LBD of ERRγ and that cadmium also mimics the effects of calcium through the LBD of ERRγ.

### Effects of calcium and cadmium on ERRγ-regulated metabolic genes in transfected HEK293T cells

**Figure 3:**
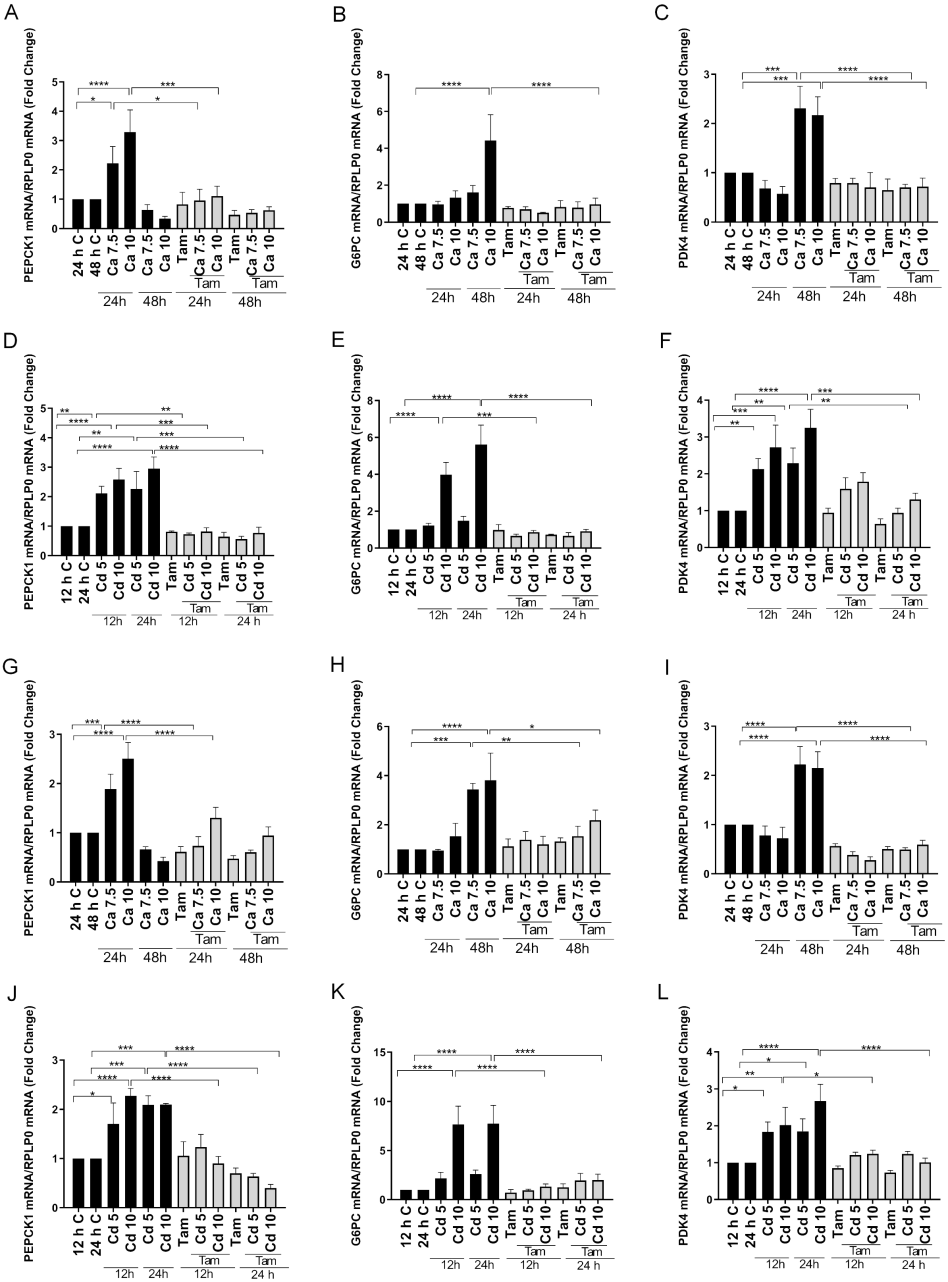
Effects of calcium and cadmium on ERRγ-regulated metabolic genes in HEK293T cells transfected with ERRγ1 or ERRγ3. HEK293T cells were transfected with ERRγ1 or ERRγ3, and treated with calcium (Ca; 7.5 or 10 mM) for 24 to 48 h or cadmium (Cd; 5 or 10 µM) for 12 to 24 h in the absence or presence of 4-hydroxy tamoxifen (Tam; 5 µM). The expression of PEPCK1, G6PC, and PDK4 was measured using a quantitative real-time qPCR assay as described in **Figure 1**. **(A–F)** Effects of calcium and cadmium on the induction of PEPCK1, G6PC, and PDK4 mRNA in HEK293T cells transfected with ERRγ1. **(G–L)** Effects of calcium and cadmium on the induction of PEPCK1, G6PC, and PDK4 mRNA in HEK293T cells transfected with ERRγ3. Data are expressed as fold change (mean ± SEM); *n* ≥ 3. Statistical significance is defined as a *p*-value of ≤0.05. *p ≤ 0.05; **p ≤ 0.01; ***p ≤ 0.001; ****p ≤ 0.0001.

**Figure 4:**
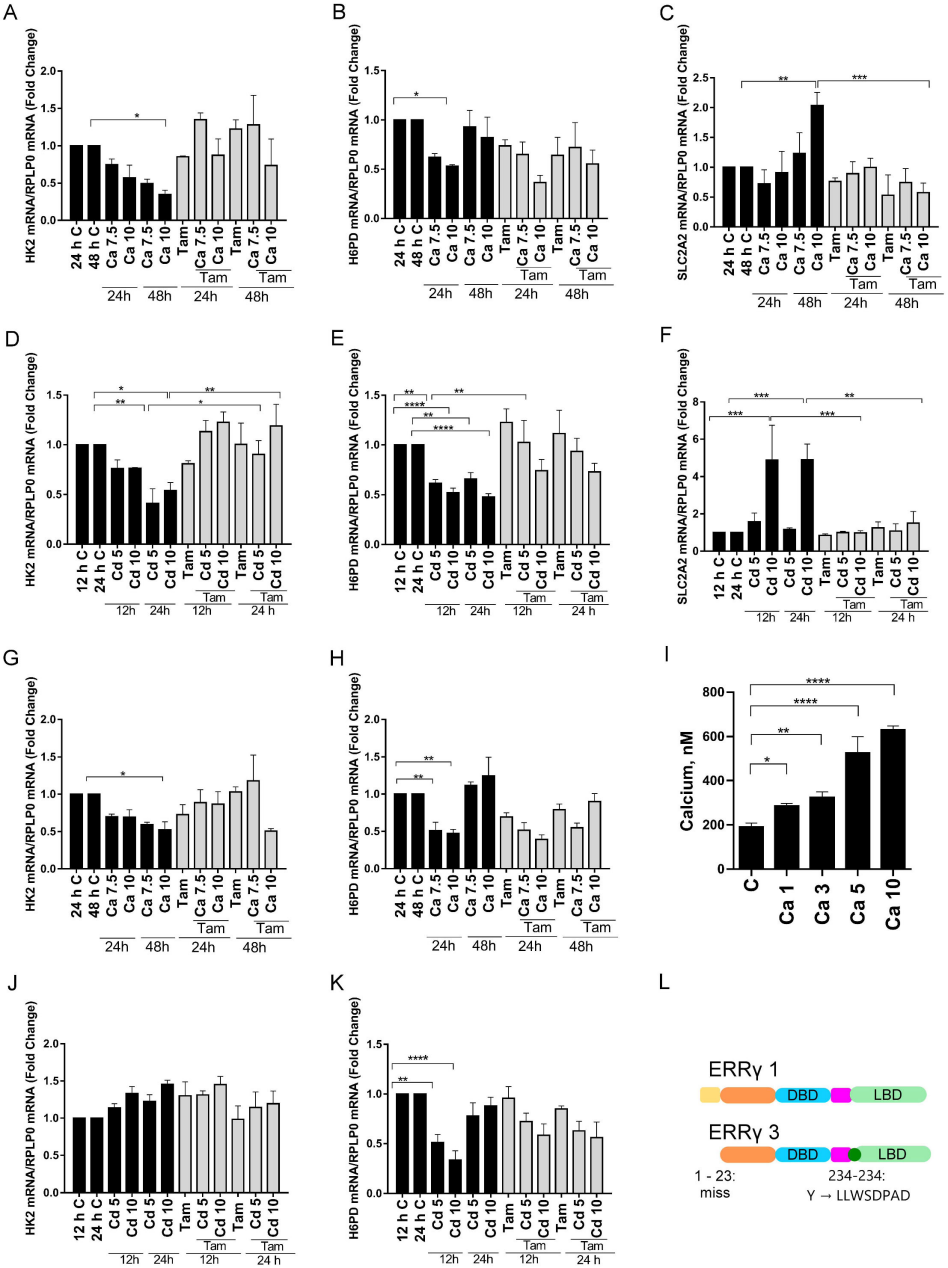
Effects of calcium and cadmium on ERRγ-regulated metabolic genes in HEK293T cells transfected with ERRγ1 or ERRγ3. Cells were treated as described in **Figure 3** and the expression of H6PD, HK2, and SLC2A2 was measured using a quantitative real-time qPCR assay as described in **Figure 1**. Cells were treated with calcium (1, 3, 5, and 10 mM) and intracellular calcium was measured as described in **Figure 1**. **(A–F)** Effects of calcium and cadmium on the induction of H6PD, HK2, and SLC2A2 mRNA in HEK293T cells transfected with ERRγ1. **(G, H, J, K)** Effects of calcium and cadmium on the induction of H6PD and HK2 mRNA in HEK293T cells transfected with ERRγ3. **(I)** Effects of extracellular calcium on the concentration of intracellular calcium. **(L)** Illustration of ERRγ1 and ERRγ3 structure. Data are expressed as fold change (mean ± SEM); *n* ≥ 3. Statistical significance is defined as a *p*-value of ≤0.05. *p ≤ 0.05; **p ≤ 0.01; ***p ≤ 0.001; ****p ≤ 0.0001.

To further demonstrate that calcium and cadmium activate ERRγ, HEK293T cells, which do not express detectable amounts of ERRγ or calcium sensing receptor and channels, were transfected with splice variants of ERRγ. The transfected cells were treated for 12, 24, or 48 hours with calcium (7.5 mM or 10 mM) or cadmium (5 μM or 10 μM) in the absence or presence of 4-hydroxy tamoxifen (5 μM). Transfection was verified by qPCR (**Supplementary Figure 1**). Similar to HepG2 cells, calcium or cadmium consistently increased the expression of PEPCK1, G6PC, and PDK4 in HEK293T cells transfected with ERRγ1 or ERRγ3. Treatment with calcium resulted in a maximal 3.2-, 4.4-, and 2.3-fold increase in PEPCK1, G6PC, and PDK4, respectively, in cells transfected with ERRγ1 (**Figures 3A–C**) and a maximal 2.5-, 3.8-, and 2.2-fold increase in PEPCK1, G6PC, and PDK4, respectively, in cells transfected with ERRγ3 (**Figures 3G–I**). Treatment with cadmium also resulted in a maximal 2.9-, 5.6-, and 3.2-fold increase in PEPCK1, G6PC, and PDK4, respectively, in cells transfected with ERRγ1 (**Figures 3D–F**) and a maximal 2.2-, 7.7-, and 2.6-fold increase in PEPCK1, G6PC, and PDK4, respectively, in cells transfected with ERRγ3 (**Figure 3J–L**). Treatment with calcium or cadmium resulted in 2.0-fold increase or 4.8-fold increase in SLC2A2 in cells transfected with ERRγ1 but had no effects on cells transfected with ERRγ3 (**Figures 4C, F**). The effects of calcium and cadmium were blocked by 4-hydroxy tamoxifen. ERRγ1 is the canonical isoform of the receptor. Compared to ERRγ1, ERRγ splice variant 3 (ERRγ3) is missing the first 23 amino acids and Y234 is replaced with LLWSDPAD. A diagram of the ERRγ splice variants 1 and 3 is shown in (**Figure 4L**). To determine whether intracellular calcium alters the activity of the ERRγ splice variants, the concentration of intracellular calcium was measured following treatment with calcium (1, 3, 5, and 10 mM) (**Figure 4I**). As expected, treatment with increasing concentrations of calcium increased the intracellular calcium concentration from 191 (±17) nM to 286 (±10) – 630 (±17) nM. Taken together, the results suggest that calcium and cadmium activate different ERRγ splice variants.

### Effects of calcium and cadmium on ERRγ-regulated metabolic genes in transfected HEK293T cells

To determine whether calcium and cadmium regulate the expression of genes in other metabolic pathways, genes in the glycolysis and pentose phosphate pathways were also measured in HEK293T cells transfected with ERRγ. Depending on the ERRγ splice variant, treatment with either calcium or cadmium had inconsistent effects on other metabolic genes (**Figure 4** and **Supplementary Figure 2**). For example, treatment with calcium decreased hexokinase 2 (HK2) in the glycolysis pathway by approximately 66% or 48% in cells transfected with ERRγ1 or ERRγ3 (**Figures 4A, G**), whereas treatment with cadmium decreased HK2 by approximately 59% in cells transfected with ERRγ1 but had no effect in cells transfected with ERRγ3 (**Figures 4D, J**). The effects of calcium and cadmium were partially blocked by 4-hydroxy tamoxifen. Treatment with calcium also decreased hexose-6-phosphate dehydrogenase (H6PD) by approximately 47% in cells transfected with ERRγ1 and 53% in cells transfected with ERRγ3 (**Figures 4B, H**). Treatment with cadmium also decreased H6PD by an approximately 53% in cells transfected with ERRγ1 and 67% in cells transfected with ERRγ3 (**Figures 4E, K**). However, the effects of calcium and cadmium were not blocked by 4-hydroxy tamoxifen.

To determine whether the effects of calcium and cadmium are specific to ERRγ, non-transfected HEK293T cells were also treated with calcium or cadmium. Treatment with calcium did not change the expression of PEPCK1 or PDK4. Treatment of cadmium did not change the expression of PEPCK1 but increased PDK4 (**Supplementary Figure 3**). PDK4 is known to be regulated by both ERRα and ERRγ, which may contribute to the increase of PDK4 (52). Non-transfected HEK293T cells have undetectable levels of G6PC and SLC2A2 (data not shown). Taken together, the results suggest that calcium and cadmium can activate different ERRγ splice variants and regulate ERRγ-responsive genes.

### Effects of calcium and cadmium on the recruitment of ERRγ to the enhancers of ERRγ target genes

**Figure 5:**
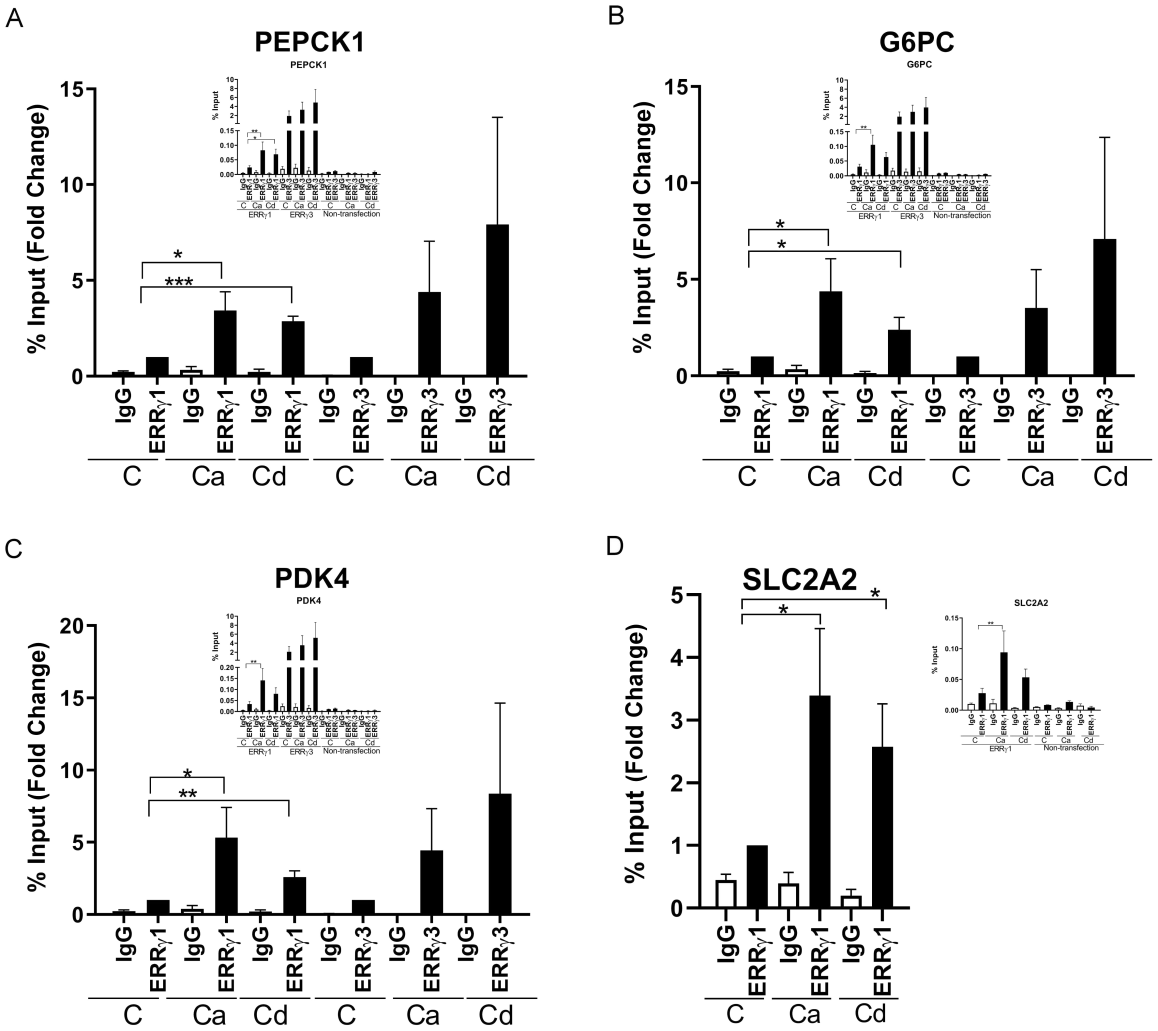
Effects of calcium and cadmium on the recruitment of ERRγ to enhancers of ERRγ-regulated genes in HEK293T cells. HEK293T cells were transfected with ERRγ1, ERRγ3, or GAL-4-ERRγ-LBD and a CAT reporter construct, and treated with calcium (Ca; 7.5 or 10 mM), cadmium (Cd; 5 or 10 µM), DY131 (1 µM), or GSK4716 (1 µM) for 1 to 48 h in the absence or presence of 4-hydroxy tamoxifen (Tam; 5 µM). The occupancy of ERRγ on the enhancer was measured using a quantitative ChIP-qPCR and CAT reporter was measured using a quantitative real-time qPCR assay as described in **Figure 2**. **(A–D)** Effects of calcium and cadmium on recruitment of ERRγ1 or ERRγ3 to the enhancer of PEPCK1, G6PC, SLC2A2, and PDK4. Data are expressed as fold change (mean ± SEM); *n* ≥ 3 except nontransfection ChIP (*n* = 2). Statistical significance is defined as a *p*-value of ≤0.05. *p ≤ 0.05; **p ≤ 0.01; ***p ≤ 0.001.

To further verify that the change of gene expression was due to the activation of ERRγ by calcium or cadmium, the recruitment of ERRγ to the enhancer regions of PEPCK1, G6PC, PDK4, and SLC2A2 was measured by a ChIP-qPCR assay in HEK293T cells transfected with ERRγ1 or ERRγ3 (**Figures 5A–D**). For HEK293T cells transfected with ERRγ1, treatment with calcium (10 mM) resulted in an approximately 3.4-, 4.3-, 5.3-, and 3.3-fold enrichment and treatment with cadmium (10 µM) resulted in a 2.8-, 2.3-, 2.5-, and 2.5-fold enrichment to the enhancer regions of PEPCK1, G6PC, PDK4, and SLC2A2, respectively. For HEK293T cells transfected with ERRγ3, treatment with calcium resulted in an approximately 4.3-, 3.5-, and 4.4-fold enrichment and treatment with cadmium resulted in a 7.9-, 7.0-, and 8.3-fold enrichment to the enhancer regions of PEPCK1, G6PC, and PDK4, respectively, and the increase was not significant. In non-transfected cells, treatment with calcium or cadmium had no effect on enrichment. The results suggest that calcium and cadmium recruit ERRγ to ERREs in the enhancer regions of target genes and induce gene expression in HEK293T cells.

**Figure 6:**
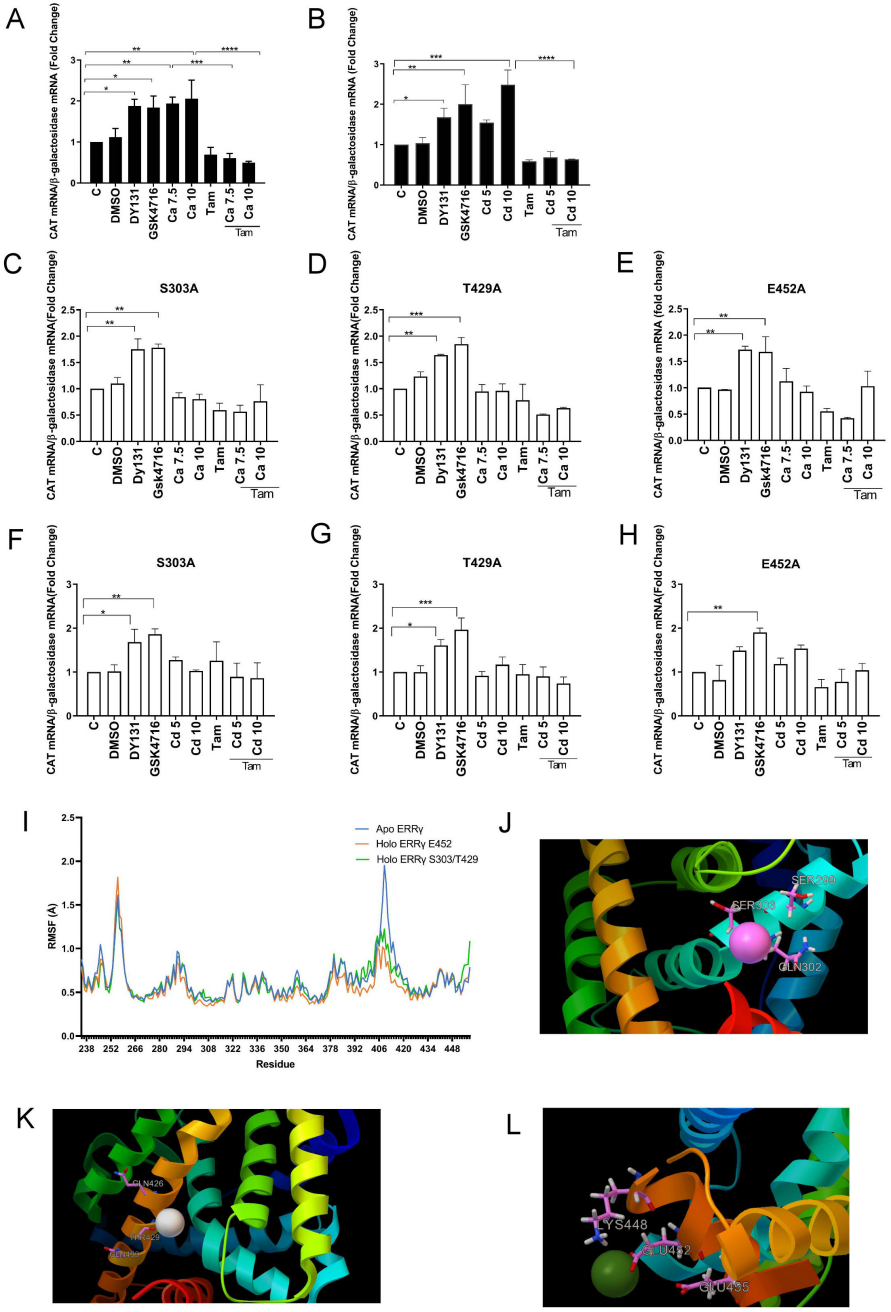
Identification of potential calcium or cadmium interaction sites on the LBD of ERRγ. HEK293T cells were transfected with GAL-4-ERRγ-LBD mutants and a CAT reporter construct and treated with DY131 (1 µM), GSK4716 (1 µM), calcium (Ca; 7.5 or 10 mM), or cadmium (Cd; 5 or 10 µM) for 24 or 48 h in the absence or presence of 4-hydroxy tamoxifen (Tam; 5 µM). The amount of CAT mRNA was measured using a quantitative real-time qPCR assay as described in **Figure 2**. **(A, B)** Effects of calcium and cadmium induction of GAL4-coupled ERRγ LBD on expression of CAT reporter. **(C–H)** Effects of calcium and cadmium on GAL-4-ERRγ-LBD mutants. Data are expressed as fold change (mean ± SEM); *n* ≥ 3. Statistical significance is defined as a *p*-value of ≤0.05. *p ≤ 0.05; **p ≤ 0.01; ***p ≤ 0.001; ****p ≤ 0.0001. **(I)** Molecular dynamics of calcium interaction with ERRγ LBD. The RMSF of the backbone atom positions was measured for the apo- and calcium-bound ERRγ LBD; *n* = 3. **(J–L)** Representative image of the interaction between ERRγ and calcium on S303, T429, and E452. Calcium and corresponding amino acid side chains are presented as ball and stick models.

To further address the mechanism by which calcium and cadmium activate ERRγ, HEK293T cells were co-transfected with GAL-4-ERRγ-LBD and a CAT reporter construct and treated with ERRγ agonists (DY131 and GSK4716), calcium, or cadmium in the absence or presence of the 4-hydroxy tamoxifen. Treatment with DY131 (1 µM) or GSK4716 (1 µM) resulted in an approximately 1.8- and 2.0-fold increase in CAT expression, respectively. Treatment with calcium (7.5 or 10 mM) for 48 h or cadmium (10 µM) for 24 h resulted in a significant increase of approximately 1.9- to 2.0- and 2.4-fold in CAT expression, respectively. Treatment with 4-hydroxy tamoxifen blocked the effects of calcium and cadmium (**Figures 6A, B**), suggesting that calcium and cadmium modulate ERRγ activity though its LBD.

### Potential interaction sites of calcium and cadmium with ERRγ

Our previous study identified four potential calcium interaction sites on the aqueous surface of the LBD of ERα (37). The first site is formed by H377, E380, and C381 on helix H4. The second site is formed by H516, N519, and E523 at the interface between helices H10 and H11. The third site is formed by K529, N532, and V534 on helix H11 and in the loop connecting helices H11 and H12 and the fourth site is formed by D538, E542, and D545 on helix H12. The high homology between the LBDs of ERα and ERRγ suggests that the corresponding sites on ERRγ are potential calcium and cadmium interaction sites. Sequence alignment identified four sites in the LBD of ERRγ (53). The first site is formed by S299, Q302, and S303 on helix H4. The second site is formed by Q426, T429, and Q433 at the interface between helices H10 and H11. The third site is formed by K439, K443, and V444 on helix H11 and in the loop connecting helices H11 and H12 and the fourth site is formed by K448, E452, and E455 on helix H12. The amino acids S303 and T429 of ERRγ have side chain groups similar to the corresponding amino acids C381 and N519 of ERα. The amino acid S303 is also conserved among the ERR family and E452 is conserved between the ERR family and ERα (**Supplementary Figures 5A–C**). To determine whether these sites are potential interaction sites of calcium and cadmium with the ERRγ, S303 on helix H4, T429 on helix H10, and E452 on helix H12 were individually mutated to alanine in the GAL-4-ERRγ-LBD, transiently transfected into HEK293T cells, and treated with calcium, cadmium, DY131, or GSK4716. Similar to wild-type GAL-4-ERRγ-LBD, treatment of GAL-4-ERRγ-LBD mutants S303A, T429A, or E452A with DY131 or GSK4716 resulted in a 1.6- to 1.7-fold or 1.6- to 1.9-fold increase of CAT, respectively, indicating that mutation of these amino acids did not alter the activity of the LBD. However, treatment of the mutants with calcium or cadmium did not significantly increase CAT expression, suggesting that S303, T429, and E452 are potential calcium and cadmium interaction sites on the ERRγ LBD (**Figures 6C–H**). The ability of DY131 and GSK4716 to activate S303A, T429A, and E452A may be due to their mechanism of activation. DY131 and GSK4716 bind in the pocket of the LBD, whereas S303, T429, and E452 are located on the aqueous surface of the LBD. For example, GSK4716 interacts with Asp328 and rearranges Arg316 and Glu275 in the pocket (54).

To further explore the interaction between calcium and the ERRγ LBD, molecular dynamics simulations were performed. To predict calcium docking sites, the crystal structure of ERRγ LBD (ID: 2GP7) was selected and its A chain representing ERRγ LBD was submitted to both metal ion-binding site for prediction: modeling server (MIB) and Autodock Vina. In agreement with the mutational analysis, MIB predicted that calcium binds to E452 and Autodock Vina predicted that calcium binds to S303 and T429. Apo ERRγ and calcium-docked ERRγ were then analyzed by molecular dynamics simulations (videos 1–3). Root mean square fluctuation (RMSF) analysis suggested that calcium binding stabilizes the LBD structure (**Figure 6J**). This effect is especially significant in the loop connecting helix H9 and helix H10 and in the N-terminus of helix H10 (amino acids 405–420) as calcium-bound ERRγ LBD has lower RMSF compared with apo ERRγ LBD. Previously published molecular dynamics simulations show that the ERRγ agonist BPA also lowers the RMSF in the same region of the LBD, suggesting that calcium mimics another ERRγ agonist to stabilize and activate ERRγ (19). The calcium-bound ERRγ molecular models are shown in **Figures 6J–L**.

### Effects of calcium and cadmium on metabolism in MCF-7 cells

**Figure 7:**
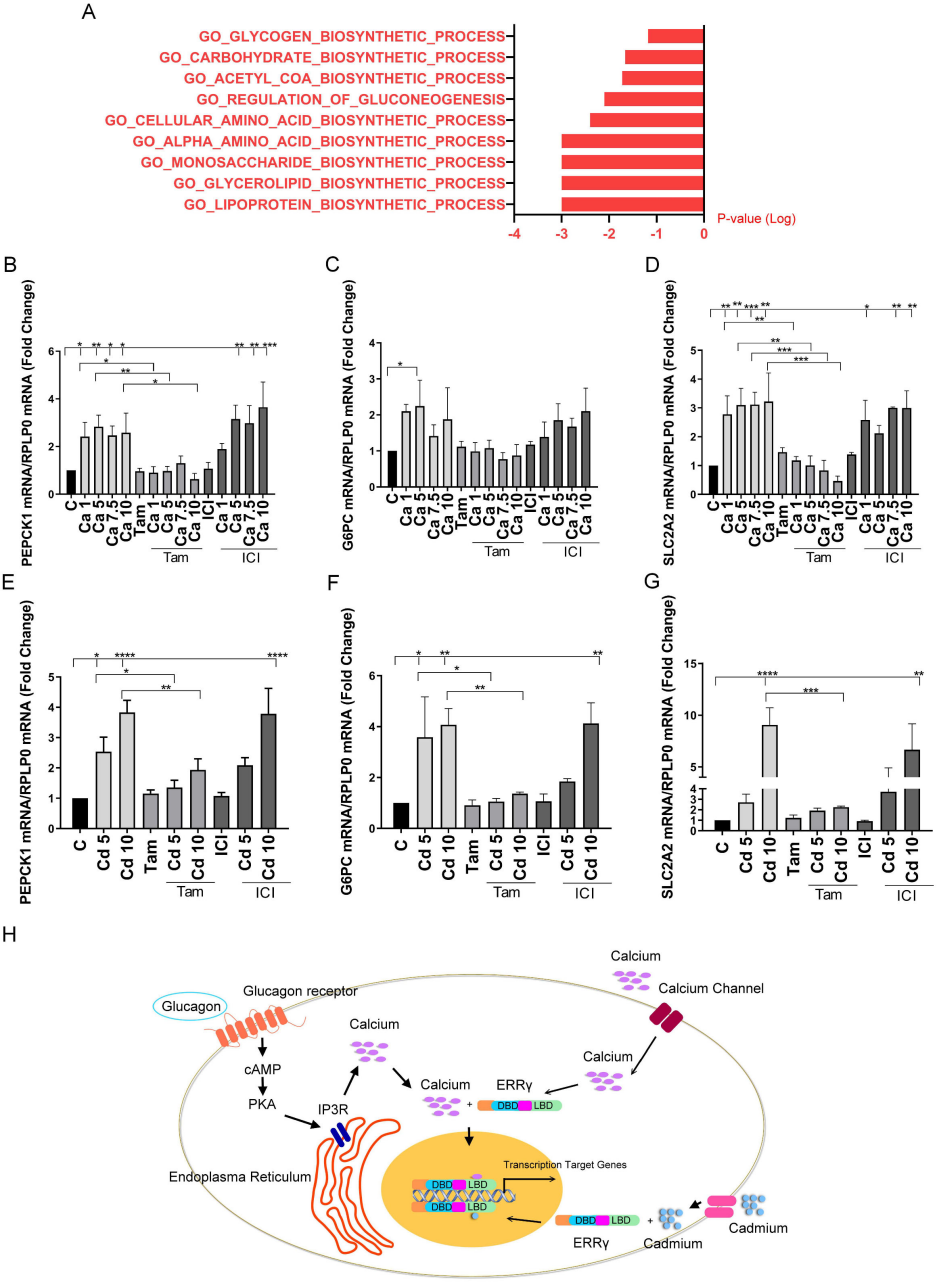
Effects of calcium and cadmium in MCF-7 cells. MCF-7 cells were treated with calcium (Ca; 1, 5, 7.5, or 10 mM) or cadmium (Cd; 5 or 10 μM) for 24 h in the absence or presence of 4-hydroxy tamoxifen (Tam; 5 μM) or ICI-182,780 (ICI; 1 μM). The expression of PEPCK1, G6PC, and SLC2A2 was measured using a quantitative real-time qPCR assay as described in **Figure 1**. Microarray results of MCF-7 treated with 1 µM cadmium were analyzed by GSEA. **(A)** “GSEA of gene expression changes in cadmium-treated MCF-7 cells. **(B–G)** Effects of calcium and cadmium on the induction of PEPCK1, G6PC, and SLC2A2 mRNA in MCF-7 cells. Data are expressed as fold change (mean ± SEM); *n* ≥ 3. Statistical significance is defined as a *p*-value of ≤0.05. *p ≤ 0.05; **p ≤ 0.01; ***p ≤ 0.001; ****p ≤ 0.0001. **(H)** Model by which calcium and cadmium mediated effects of glucagon. Glucagon activates ERRγ through IP3R-induced increase in intracellular calcium. Calcium, in turn, activates ERRγ through its LBD. Extracellular calcium through calcium channels also activates ERRγ. Cadmium mimics calcium to activate ERRγ through its LBD.

To better understand the role of calcium and cadmium in influencing metabolic genes, we re-analyzed our previously published microarray results using GSEA. In that study, MCF-7 cells were treated with cadmium and microarrays were used to measure gene expression (11). As expected, GSEA showed a significant increase of hallmark estrogen response early and late pathways (data not shown), which is consistent with our previously published studies showing that cadmium binds to and activates ERα. GSEA also identified an increase in several biosynthetic pathways, including an increase in the carbohydrate biosynthetic pathway (**Figure 7A**), suggesting that cadmium influences metabolism in MCF-7 cells. To verify the microarray results, MCF-7 cells were treated with calcium (1, 3, 5, and 10 mM) or cadmium (5 or 10 µM) in the absence or presence of 4-hydroxy tamoxifen (5 μM) or the ERα antagonist ICI-182,780 (fluvestrant, 1 μM). Similar to HepG2 cells and ERRγ-transfected HEK293T cells, treatment with calcium resulted in a maximal 2.8-, 2.2-, and 3.2-fold increase in PEPCK1, G6PC, and SLC2A2, respectively (**Figures 7B–D**) and treatment with cadmium resulted in a maximal 3.8-, 4.0-, and 9.0-fold increase in PEPCK1, G6PC, and SLC2A2, respectively (**Figures 7E–G**). The effects of both calcium and cadmium were blocked by 4-hydroxy tamoxifen. To verify that the effects of calcium and cadmium were not due to the activation of ERα, MCF-7 cells were also treated with ICI-182,780. The antiestrogen did not significantly block the effects of calcium or cadmium, suggesting that metal activation of ERα was not responsible for the increase in PEPCK1, G6PC, and SLC2A2. Lastly, immunofluorescence verified the expression of ERRγ in MCF-7 cells (**Supplementary Figure 4**). Taken together, the results suggest that calcium and cadmium increase the expression of genes involved in gluconeogenesis through the activation of ERRγ.

## Discussion

Although disruption of metabolism is linked to many diseases such as obesity, type 2 diabetes, nonalcoholic fatty acid liver disease, and several cancers, the underlying mechanisms responsible for metabolic disruption are largely unknown (1–4). ERRγ is a key regulator of genes involved in gluconeogenesis. It is a member of the superfamily of nuclear receptors but, unlike many members of the superfamily, ERRγ has no known physiological ligand (19, 21). The present study suggests that calcium is a potential ligand of ERRγ. More specifically, the results show that calcium mediates the effect of glucagon on gluconeogenesis and that cadmium, which mimics calcium, disrupts metabolism through ERRγ. The results also show that calcium and cadmium activate ERRγ through the LBD of the receptor and identifies S303, T429, and E452 as potential interaction sites **Figure 7H**.

Similar to other nuclear receptors, members of the ERR family have an N-terminus transactivation domain, a DNA binding domain, a hinge region, and a C-terminus LBD (22). The ERRs share high structural and sequence homology (34). However, key residues in the LBD discriminate between binding of a specific ligand. For example, BPA is a synthetic ligand of ERRγ but has weak affinity for ERRβ and no affinity for ERRα. Although the binding pockets of ERRγ and ERRβ accommodate BPA, the amino acid N346, which is unique to the LBD of ERRγ, strengthens the binding of BPA, resulting in a higher affinity of ERRγ for BPA (55). In contrast to ERRγ and ERRβ, the bulky amino acids F232 and F286 in the LBD of ERRα prevent the binding of BPA. The LBD of ERRγ has the typical three-layered α-helix sandwich structure composed of 11 α-helices (H1 and H3–H12) (53). One well-studied nuclear receptor is retinoid X receptor (RXR). Based on the crystal structure of the apo and holo LBD of RXRα, binding of ligand results in several major structural changes. Helix H11 rotates around its axis by approximately 180° to generate a contiguous helix with helix H10 to form the dimerization domain. Helix H3 rotates around its axis by approximately 90° and helix H12 is repositioned to seal the ligand-binding pocket of LBD of RXRα to form the coactivator binding site (56). Similar to RXR, the binding of synthetic compounds alters the structure of ERRγ. For example, the agonist GSK4716 relocates helices H1–H3 to enlarge the cavity of the LBD, allowing it to fit in the pocket. The antagonist 4-hydroxy tamoxifen dislocates helices H11 and H12 to block the binding of coactivator, thus inhibiting ERRγ transcriptional activity (53, 57). In contrast to compounds that alter the structure of ERRγ by binding in the pocket, coregulators and some synthetic compounds such as BPA have a minor influence on the structure of ERRγ and, instead, stabilize the structure of ERRγ to increase its activity (57). For example, peroxisome proliferator-activated receptor-gamma coactivator 1 alpha (PGC1-α), which functions as an ERRγ coactivator, stabilizes the ERRγ structure by reducing the kinetics of helices H6, H7, H10/H11, and H12 (58). The molecular dynamics simulation of BPA-bound ERRγ shows that regions such as the loop connecting helix H3 and helix H4 and the loop connecting helix H9 and helix H10 have a lower RMSF, suggesting that these regions are rigidified in BPA-bound ERRγ, which stabilizes the LBD (19). The present study identified S303 on helix H4, T429 on helix H10, and E452 on helix H12 as potential calcium and cadmium interaction sites with ERRγ. Similar to the molecular dynamics simulations with BPA, the binding of calcium to ERRγ lowered the RMSF of the loop connecting helix H9 and helix H10 and the N-terminus of helix H10, suggesting that calcium binding to these sites stabilizes the ERRγ structure in a manner similar to BPA. In the simulation, calcium docked to S303 but escaped from the water cage and when calcium docked to T429, it migrated and docked to S303. Interestingly, the location of the calcium and cadmium interaction sites are also located close to functional regions in ERRγ. A recent study shows that the ERRγ homodimer is formed by an interaction between helices H10. The amino acid pairs P422–P422, R425–Q426, and Q426–R425 are essential to form intermolecular interactions at the homodimer interface (59). A calcium and cadmium interaction site at T429 on helix H10 is close to this region, suggesting that T429 may be involved in the formation of the dimerization domain of ERRγ. In the case of the calcium and cadmium interaction site at E452 on helix H12, calcium binding to E452 of ERRγ in our molecular dynamics simulations and PGC-1α binding to the ERRγ in a previously published paper show that these interactions stabilize the structure of ERRγ, suggesting that calcium interaction with E452 may have effects similar to PGC-1α (58). In addition to molecular dynamics simulations, sequence alignment of the LBD of ERRγ and ERα shows that the amino acids of the calcium and cadmium interaction sites share similar side chain properties to the calcium interactions sites in ERα. For example, the amino acid S303 of ERRγ and the corresponding amino acids C381 of ERα are nucleophilic. S303 is also conserved among the ERR family. The amino acid T429 of ERRγ has a polar side chain group similar to the corresponding amino acid N519 of ERα. E452 is conserved between the ERR family and ERα. Taken together, these data suggest that the potential interaction sites of calcium in ERRγ are important and require further investigation. Although the data suggest a model whereby metals interact with sites on the surface of the LBD, we cannot rule out an indirect effect of the metals. This proposed model remains to be validated.

The DNA and LBDs of ERRα and ERRγ are highly conserved while their N-terminal activation domains are less conserved. Because of the high homology of their DNA binding domains, ERRα and ERRγ target the same ERRE and have overlapping regulatory effects on metabolic genes (18–20). Moreover, some studies show that ERRα and ERRγ can form heterodimers (33–35). For example, ERRα and ERRγ enhance aerobic glycolysis by binding to ERREs in the promoter region of glycolytic genes (34, 60). However, ERRγ increases the expression of PEPCK while ERRα inhibits the expression of PEPCK, suggesting that ERRγ and ERRα have opposing regulatory effects on the gluconeogenesis pathway (34, 35, 61). HK2 and H6PD are also regulated by ERRα and ERRγ (20, 60). In this study, treatment of HEK293T cells transfected with ERRγ with calcium and cadmium resulted in a decrease in the expression of HK2 and H6PD that was not blocked by 4-hydroxy tamoxifen. It is possible that heterodimerization of ERRγ with ERRα influences the effects of 4-hydroxy tamoxifen. Whether ERRγ and ERRα function as a heterodimer and whether heterodimers are agonistic or antagonistic are largely unknown. The interaction between ERRα and ERRγ and their influence on global metabolic regulation still require more investigation.

Although there are only a few studies, the expression of ERRγ splice variants appears to be tissue specific (32). The functions of different ERRγ splice variants are largely unknown. In this study, two ERRγ isoforms were studied. Compared to ERRγ1, ERRγ3 is missing the first 23 amino acids and Y234 in the N-terminus of the LBD is replaced with LLWSDPAD. Interestingly, many ERRγ splice variants are missing the first 23 amino acids. One study shows that the AF-1 of ERRγ1 recruits coactivators while the AF-1 of ERRγ2, which is missing the first 23 amino acids, does not recruit coactivators, suggesting that the first 23 amino acids is an additional N-terminal AF-1 (62). Similar results are observed for other nuclear receptors. Hepatocyte nuclear factor 4α7 and hepatocyte nuclear factor 4α1 also differ in the N-terminus. Hepatocyte nuclear factor 4α7 is missing the first 13 amino acids and has no measurable AF-1 activity (63). Compared to progesterone receptor B, progesterone receptor A is missing the first 164 amino acids. Progesterone receptor B has a transcription activation domain 3 (AF-3) in the N terminus (amino acids 54–154) that synergizes with AF-1 and AF-2 (64, 65). Although the effects of the insertion between the hinge region and the LBD of ERRγ are not known, previous studies suggest that insertions in the LBD of nuclear receptors have different regulatory effects. For example, the human constitutive androstane receptor splice variant 2 has an additional nine amino acids in the LBD that is associated with loss of transcriptional activity (66). Estrogen receptor β isoform 2 in rat has an additional 18 amino acids between helix H5 and H6 in the LBD. Estrogen receptor β isoform 2 is not active and inhibits the transcriptional activity of ERα and estrogen receptor β isoform 1 (67). In this study, treatment of HEK293T cells transfected with ERRγ1 or ERRγ3 with calcium and cadmium increased the expression of PEPCK1 and G6PC, suggesting that genes in gluconeogenesis are regulated by ERRγ splice variants 1 and 3. In contrast to PEPCK1 and G6PC, SLC2A2 was upregulated by ERRγ1 but not by ERRγ3, suggesting that sequences of ERRγ splice variants influence the effects of ERRγ. Moreover, treatment with calcium and cadmium increased the expression of PDK4 in HepG2 cells and HEK293T cells transfected with ERRγ but not in MCF-7 cells (data not shown), suggesting that the regulatory effects of ERRγ in different tissues may be due to different splice variants and/or coregulators. These results suggest that ERRγ splice variants have different regulatory effects.

In the body, calcium has important physiological functions including signaling in cell proliferation and stemness, muscle contraction, and hormone secretion (13). In addition, calcium facilitates protein processing and activates other proteins such as calcineurin (13, 49). Therefore, calcium homeostasis is essential and alternation of calcium homeostasis results in several pathological conditions especially metabolic disease (12–15). On the other hand, cadmium is a heavy metal with no physiological function that accumulates in the body (68). Cadmium mimics calcium due to its similar charge and ionic radius, but has a higher affinity for some proteins than calcium. Because cadmium has a higher affinity for some proteins, it disrupts normal protein function and signaling pathways. Several studies have linked cadmium with obesity and type 2 diabetes (9, 10). Furthermore, several studies have identified cadmium as a human pancreatic carcinogen (69–71). Although several epidemiologic studies have explored the relationship between metabolic diseases, such as obesity and diabetes, and calcium and cadmium (9, 10, 72–74), the mechanisms that are responsible for the effects of calcium and cadmium in metabolic diseases are not well understood (9, 10, 72–74). This study shows that calcium is a potential ligand of the metabolic regulator ERRγ that mediates the effects of glucagon on genes in the gluconeogenesis pathway and that cadmium, which mimics calcium, disrupts metabolism through ERRγ, providing a possible mechanism for the effects of calcium and cadmium in metabolic diseases.

### Limitations of the study

This study has revealed that calcium is a potential ligand of ERRγ that mediates the effects of glucagon and that cadmium, which mimics the effects of calcium, disrupts metabolism through ERRγ. While this study identified three potential interaction sites (S303, T429, and E452), other interaction sites and mechanisms of activation remain undetermined. Moreover, this study raised new questions about the role of calcium and cadmium in metabolism. In pursuit of answering these questions, future research should involve *in vivo* studies and structural crystallography studies.

**Table 1:** Primer sequence and taqman probe catalog number.

Primer sequence		
Experiment	Gene	Primer sequence
Chip	PEPCK1	Forward: TTGAGACCTAGCCCC GTTCT
		Reserve: AGTTTAGGGAGTGGAGGGGG
	G6PC	Forward: ACACCAAGGGCTGTAACTTT
		Reverse: TGGTTTGGGAGATCAACCCC
	PDK4	Forward: TAGACGAAGAGGGCAAAGGA
		Reverse: TGGTGAAGATGGGGTTTCTC
	SLC2A2	Forward: GCAAAAGGTCATAGCATGGTCA
		Reverse: ACCTGGTCACTGAGTATGGTGA
Mutagenesis	S303A	Forward: GAATCTCCATCCATGCAGCC TGGAGGAGGCTCATCT
		Reverse: AGATGAGCCTCCTCCAGGCTG CATGGATGGAGATTC
	T429A	Forward: TGGACTGCCTTGGCGGAGGTC TGCCTC
		Reverse: GAGGCAGACCTCCGCCAAGGCA GTCCA
	E452A	Forward: CTTGGCCTCCAGCATTGCCAA AAAAAGTTTGTGCATGGG
		Reverse: CCCATGCACAAACTTTTTTTG GCAATGCTGGAGGCCAAG
CAT reporter	CAT	Forward: TTCTTGCCCGCCTGATGAAT
		Reverse: ACCGTAACACGCCACATCTT
	β-galactosidase	Forward: ATGGGTAACAGTCTTGGCGG
		Reverse: GGCGTATCGCCAAAATCACC
Taqman probe catalog		
Gene	Catalog	
ERRγ	Hs00976243_m1	
PEPCK1	Hs01572978_g1	
G6PC	Hs00609178_m1	
PDK4	Hs01037712_m1	
SLC2A2	Hs01096908_m1	
HK2	Hs00606086_m1	
H6PD	Hs00188728_m1	
MDH2	Hs00606086_m1	
LDHA	Hs01378790_g1	
SLC2A1	Hs00892681_m1	
PFKL	Hs01036347_m1	
PK	Hs00987255_m1	
RPLP0	Hs00420895_gH	

## Data Availability

The raw data supporting the conclusions of this article will be made available by the authors, without undue reservation.

## Glossary

DAPI: 4, 6-diamidino-2-phenylindole
PFKL: 6-phosphofructokinase, liver type
BPA: bisphenol A
CCS: charcoal stripped calf serum
CAT: chloramphenicol acetyltransferase
ChIP: chromatin immunoprecipitation Assay
DBD: DNA binding domain
DMEM: Dulbecco's Modified Eagle's Medium
ERRγ1: ERRγ splice variant 1
ERRγ2: ERRγ splice variant 2
ERRγ3: ERRγ splice variant 3
ERα: estrogen receptor α
ERRγ: estrogen-related receptor γ
ERR: estrogen-related receptor
ERRE: estrogen-related response elements
FBS: fetal bovine serum
GSEA: gene set enrichment analysis
G6PC: glucose-6-phosphatase
HBSS: Hanks' balanced salt solution
HK2: hexokinase 2
H6PD: hexose-6-phosphate dehydrogenase
IMEM: improved minimal essential medium
IP3R: inositol 1,4,5 trisphosphate receptor
LDHA: lactate dehydrogenase A
LBD: ligand-binding domain
MDH2: malate dehydrogenase 2
PGC-1α: peroxisome proliferator-activated receptor-gamma coactivator 1α
PEPCK: phosphoenolpyruvate carboxykinase
PDK4: pyruvate dehydrogenase kinase 4
PK: pyruvate kinase
RIPA: radioimmunoprecipitation assay
real-time qPCR: real-time quantitative polymerase chain reaction
RPLP0: ribosomal protein P0
RMSF: root mean square fluctuation
SLC2A1: solute carrier family 2 member 1
SLC2A2: solute carrier family 2 member 2
