# Supplementary material for: Activation of estrogen-related receptor γ by calcium and cadmium
Source: Front Endocrinol (Lausanne). 2024 Oct 23;15:1400022. doi: 10.3389/fendo.2024.1400022 (PMC11537906; doi:10.3389/fendo.2024.1400022)
Supplement: Supplementary file 1 [file DataSheet1.pdf]

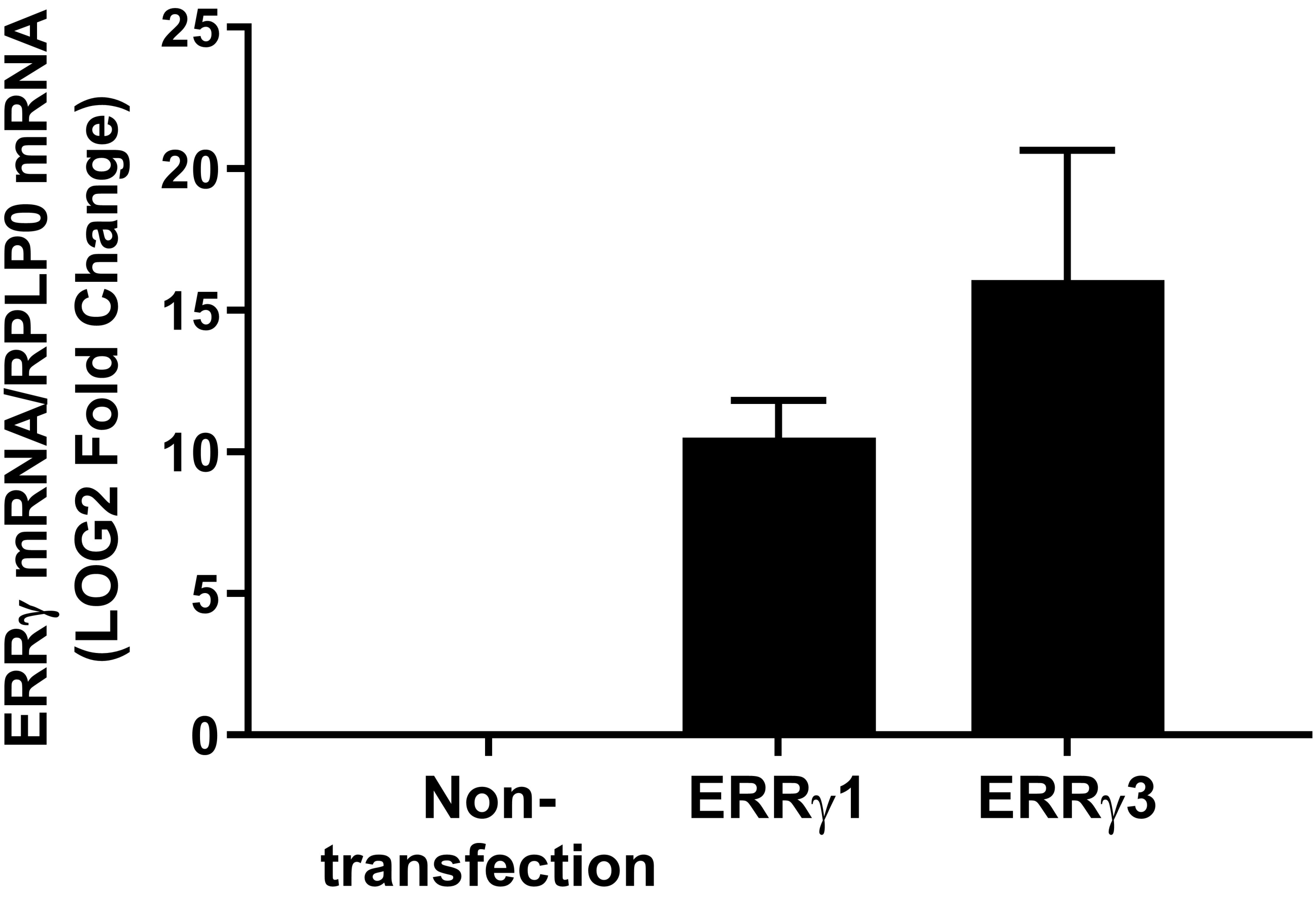
ERRγ mRNA/RPLP0 mRNA
(LOG2 Fold Change)
25
20
15
10
5
0
Non-transfection
ERRγ1
ERRγ3

Supplement figure 1. The transfection of ERRγ1 or ERRγ3 in HEK293T cells. HEK293T cells were transfected with *ERRγ1* or *ERRγ3*, and *ERRγ* expression was verified by a quantitative real time qPCR as described in Figure 1.

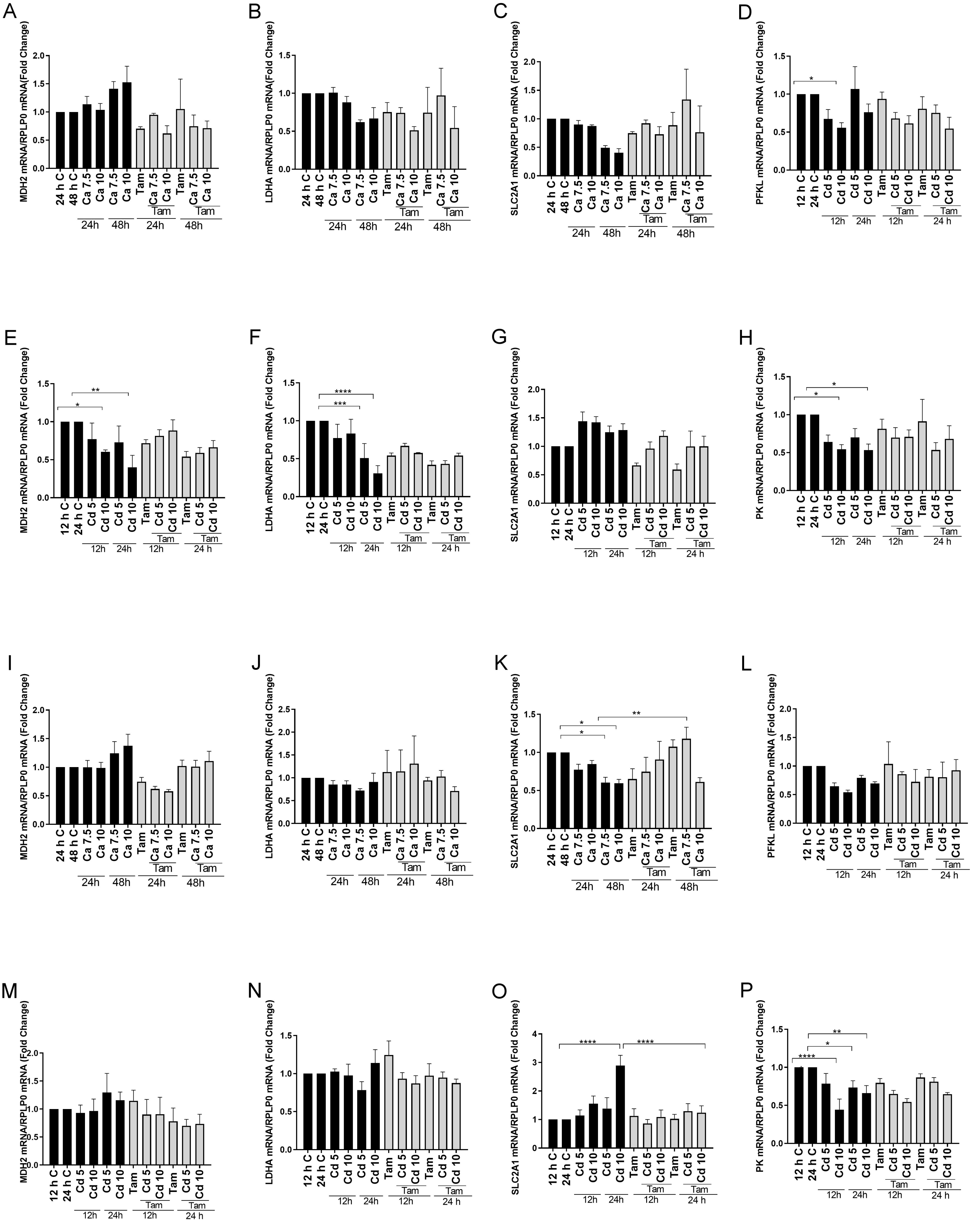

A
MDH2 mRNA/RPLP0 mRNA(Fold Change)
B
LDHA mRNA/RPLP0 mRNA(Fold Change)
C
SLC2A1 mRNA/RPLP0 mRNA (Fold Change)
D
PFKL mRNA/RPLP0 mRNA (Fold Change)
E
MDH2 mRNA/RPLP0 mRNA (Fold Change)
F
LDHA mRNA/RPLP0 mRNA (Fold Change)
G
SLC2A1 mRNA/RPLP0 mRNA (Fold Change)
H
PK mRNA/RPLP0 mRNA (Fold Change)
I
MDH2 mRNA/RPLP0 mRNA (Fold Change)
J
LDHA mRNA/RPLP0 mRNA (Fold Change)
K
SLC2A1 mRNA/RPLP0 mRNA (Fold Change)
L
PFKL mRNA/RPLP0 mRNA (Fold Change)
M
MDH2 mRNA/RPLP0 mRNA (Fold Change)
N
LDHA mRNA/RPLP0 mRNA (Fold Change)
O
SLC2A1 mRNA/RPLP0 mRNA (Fold Change)
P
PK mRNA/RPLP0 mRNA (Fold Change)

Supplement figure 2. Effect of calcium and cadmium on metabolic genes in HEK293T cells transfected with ERRγ1 or ERRγ3. HEK293T cells were transfected with *ERRγ1* (A-H) or *ERRγ3* (I-P), and treated with calcium (7.5 mM or 10 mM) or cadmium (5 µM or 10 µM) in the presence or absence of 4-hydroxy tamoxifen (5 µM) for 12 hours to 48 hours. The expression of malate dehydrogenase 2 (*MDH2*), lactate dehydrogenase A (*LDHA*), solute carrier family 2 member 1 (*SLC2A1*), 6-phosphofructokinase, liver type (*PFKL*), and pyruvate kinase (*PK*) were measured by quantitative real time qPCR as described in Figure 1. Data are expressed as fold change (mean ± SEM); n≥3; Statistical significance is defined as a P value of ≤ 0.05. *p≤0.05; **p≤0.01; ***p≤0.001; ****p≤0.0001.

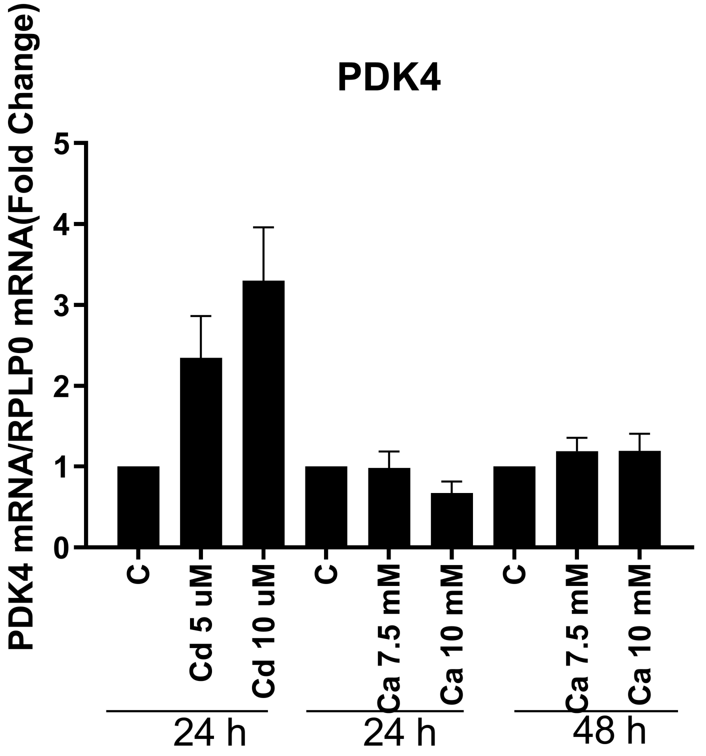
PDK4
PDK4 mRNA/RPLP0 mRNA(Fold Change)
0
1
2
3
4
5
C
Cd 5 uM
Cd 10 uM
C
Ca 7.5 mM
Ca 10 mM
C
Ca 7.5 mM
Ca 10 mM
24 h
24 h
48 h

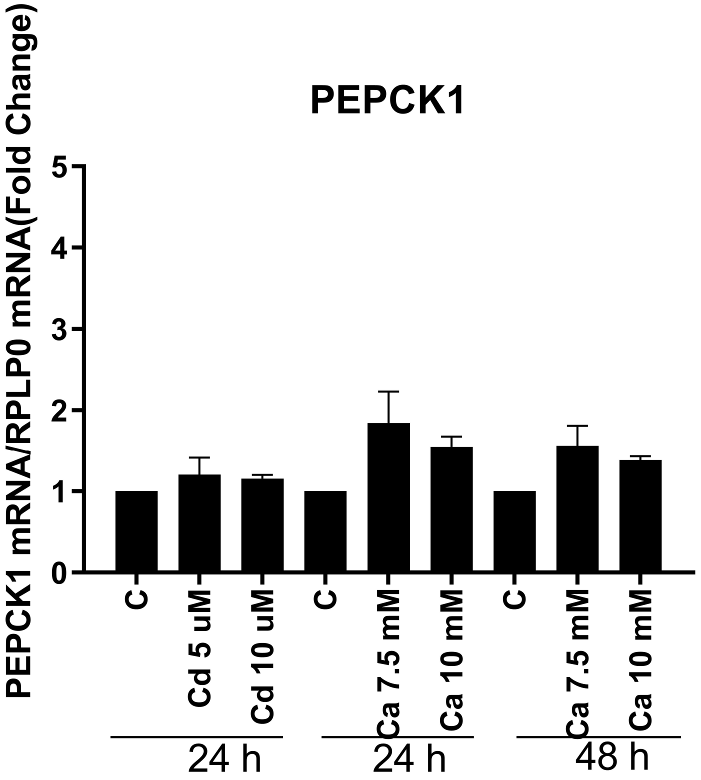
PEPCK1
PEPCK1 mRNA/RPLP0 mRNA(Fold Change)
0
1
2
3
4
5
C
Cd 5 uM
Cd 10 uM
C
Ca 7.5 mM
Ca 10 mM
C
Ca 7.5 mM
Ca 10 mM
24 h
24 h
48 h

Supplement figure 3. Effect of calcium or cadmium in non-transfected HEK293T cells. HEK293T cells were treated with calcium (7.5 mM or 10 mM) for 24 hours to 48 hours, or cadmium (5 μM or 10 μM) for 24 hours. The expression of *PDK4* and *PEPCK1* was measured by quantitative real time qPCR as described in Figure 1. Data are expressed as fold change (mean ± SEM).

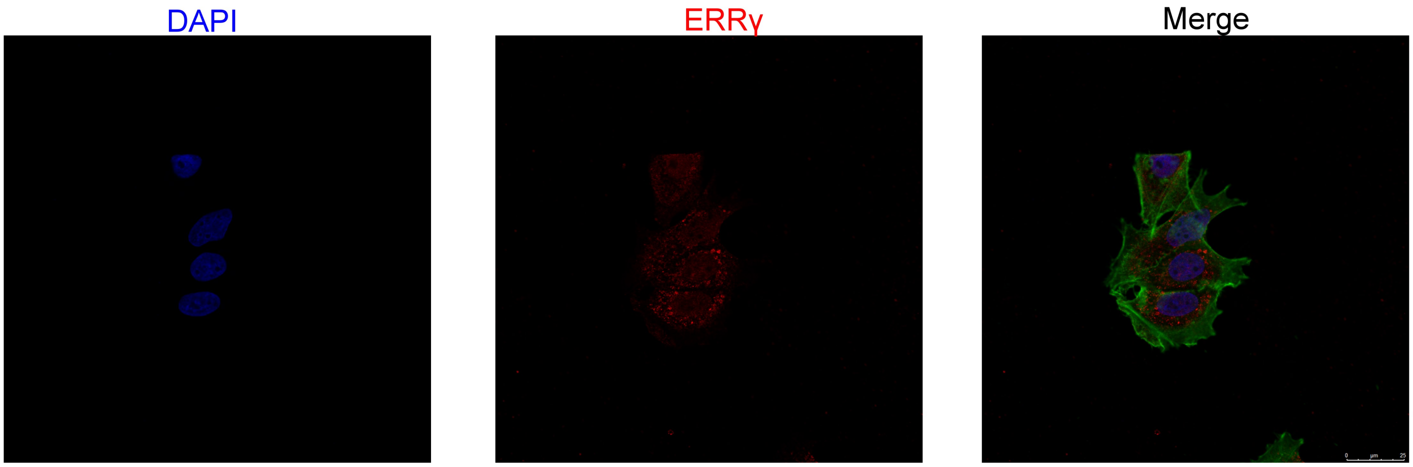
DAPI
ERRγ
Merge

Supplement Figure 4. Immunofluorescence detection of ERRγ in MCF-7. Immunofluorescence was described in Figure 2.

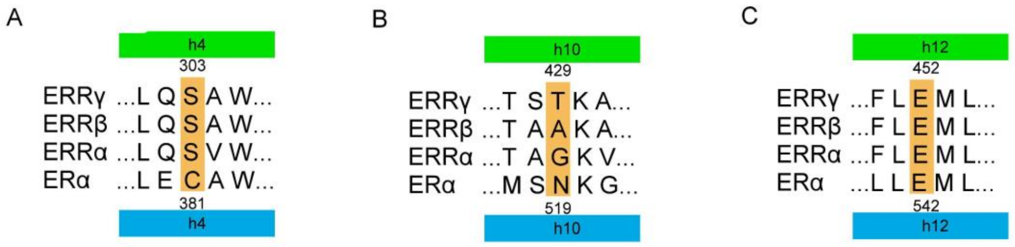
A
h4
303
ERRγ ...L Q S A W...
ERRβ ...L Q S A W...
ERRα ...L Q S V W...
ERα ...L E C A W...
381
h4
B
h10
429
ERRγ ...T S T K A...
ERRβ ...T A A K A...
ERRα ...T A G K V...
ERα ...M S N K G...
519
h10
C
h12
452
ERRγ ...F L E M L...
ERRβ ...F L E M L...
ERRα ...F L E M L...
ERα ...L L E M L...
542
h12

Supplement figure 5. The interaction sites of calcium and cadmium with ERRγ. The alignment of the LBD of ERα and ERRs are illustrated. Green represents the helices of ERRγ LBD, blue represents the helices of ERα LBD, and yellow highlights the corresponding sites of ERRγ and ERα that interact with calcium or cadmium. The number represents the exact location of amino acid.
